# Emerging multifunctional polymer binder materials for advancing lithium–sulfur battery performance: a review

**DOI:** 10.1039/d6ra03979k

**Published:** 2026-07-14

**Authors:** Pranjalee Ghosh, Manu U. M. Patel

**Affiliations:** a Manipal Institute of Technology, Manipal Academy of Higher Education Manipal India manu.patel@manipal.edu

## Abstract

Lithium–sulfur (Li–S) batteries have emerged as a strong next-generation energy storage technology, mainly due to their high theoretical energy density and low cost. However, their commercialization has not been possible due to the intrinsically low conductivity of sulfur, severe volume expansion and polysulfide shuttling during cycling, leading to rapid capacity fading and low coulombic efficiency. While extensive research is focused on the development of advanced cathode hosts and electrolyte optimization, the importance of polymer binders in stabilizing Li–S cathodes has only recently been recognized as a crucial direction to further improve the performance of Li–S batteries. This review provides a comprehensive overview of recent progress in polymer binder engineering for Li–S battery cathodes. The review highlights the transition from conventional inert binders to multifunctional systems. Herein, we discuss different types of multifunctional binders, including polar binders for polysulfide confinement, conductive binders for improved charge transport and crosslinked or elastomeric networks for enhanced mechanical durability. Emerging approaches, including supramolecular, self-healing, bio-based, water-processable and binder-free strategies, are also critically evaluated. By correlating binder chemistry and architecture with electrochemical performance, this review outlines the key design principles and their advantages, limitations and future directions towards practical, high-performance and scalable Li–S battery technologies.

## Abstract 2

From inert binders to multifunctional systems: this review explores how polymer binder engineering improves polysulfide regulation, electrode integrity, and electrochemical performance in Li–S batteries.

## Graphical abstract

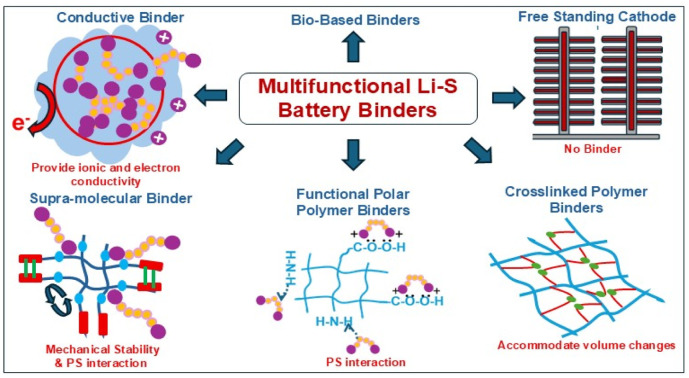

## 1. Introduction

Global energy demand has seen a rapid increase in the past few decades, mainly due to population growth and industrialization. This increase in energy demand has resulted in environmental damage and accelerated climate change, largely due to continued consumption of fossil fuels. Addressing these challenges requires a transition toward sustainable and renewable energy sources. The use of solar, wind and tidal power, along with the development of efficient and reliable energy storage technologies, is very much the need of the hour.^1,2^ Meeting this growing demand for renewable energy integration requires advanced energy storage technologies capable of delivering high energy density, large capacity and long-term electrochemical stability. Conventional lithium-ion (Li-ion) batteries are approaching their practical limits in terms of energy density and capacity, rendering them insufficient for future large-scale energy applications.^3–5^ Consequently, significant research efforts have been directed toward alternative battery chemistries with substantially higher theoretical energy densities and capacities. Lithium–air, zinc–air, aluminum–air and lithium–sulfur (Li–S) batteries have emerged as promising candidates due to their exceptionally high theoretical energy densities and capacities.^6–8^

Li–S batteries have emerged as one of the most prominent new-generation batteries due to their high theoretical energy density (2600 Wh kg^−1^) and capacity (1675 mAh g^−1^) and low material cost, considering the natural abundance of sulfur (S). Despite these advantages, their commercialization has been delayed due to (i) poor conductivity of the active material S and lithium sulfide (Li_2_S), (ii) large volume expansion (up to ≈80%) during cycling, (iii) dissolution and migration of lithium polysulfides (polysulfide shuttling) and (iv) mechanical degradation of the cathode.^9–11^

To address these challenges faced by Li–S batteries, researchers have adopted a multi-dimensional approach. This includes using different conductive carbon and other materials in the cathode to overcome the insulating nature of S and Li_2_S and encapsulate the lithium polysulfide (PS) intermediates. Electrolyte additives are designed to protect the lithium anode. Ionic liquids and polymer electrolytes are considered to mitigate PS shuttling. Modified separators are used to block the shuttling of PS between the electrodes. These approaches have significantly helped to address the challenges associated with Li–S batteries by extending their cycle-life and improving performance.^12–20^ However, these strategies have also resulted in lower energy density and higher cost due to the use of additional battery components.^21^

Polymer binders, although present in a small fraction in the Li–S cathode (5–10 wt%), play a very important role in ensuring optimal battery performance. Their importance in the Li–S electrode fabrication has been increasingly recognized, with numerous studies demonstrating significant improvements in the battery performance arising from the advances in polymer binders.^21–25^

Herein, we systematically present recent advances in Li–S cathode binder engineering with emphasis placed on multifunctional designs that extend beyond conventional mechanical adhesion requirements. Specifically, this work covers functional polar polymer binders that chemically anchor lithium PS's, crosslinked and elastomeric networks that accommodate large volume changes and preserve electrode integrity. Electron-conductive, lithium-ion conductive binders and their working mechanisms resulting in improved battery performance are discussed. Emerging technologies, such as supramolecular and dynamic covalent binders that are capable of self-healing and facilitating stress relaxation, are discussed for their ability to maintain the electrode integrity during repeated cycling. This review gives an overview of binder-free cathode designs, particularly in terms of PS confinement and enhanced reaction kinetics. Bio-derived and water-processable binders that enable sustainable and scalable alternatives are also discussed.

This review correlates the binder chemistries and structural designs with their functional working mechanisms and their influence on the electrochemical performance. It highlights the relationships influencing the electrode stability and active material loading, utilization and battery performance. Overall, this review provides an update on the latest developments leading to the evolution of advanced multifunctional binders that are enabling optimized Li–S cathodes.

## 2. Components of Li–S batteries and their roles in battery operation

Li–S batteries are composed of four primary components: the anode, cathode, electrolyte and separator, each playing a distinct role in energy storage and delivery. Anode (lithium metal): the anode typically consists of metallic lithium, which serves as the source of lithium ions. During discharge, lithium atoms release electrons and form lithium ions. The electrons travel through the external circuit to power a device, while lithium ions migrate through the electrolyte toward the cathode. Cathode (carbon-/S-based composite): the cathode contains elemental S or Li_2_S embedded within a conductive matrix (usually carbon-based materials). S acts as the active material that stores and releases energy through an electrochemical redox conversion reaction. Electrolyte: the electrolyte is a liquid or gel medium that allows lithium ions to transit between the anode and cathode. In Li–S batteries, the electrolyte plays a more complex role because the intermediate PS formed during cycling are soluble in common electrolytes, which can lead to unwanted side reactions.^8–13^

**Fig. 1:**
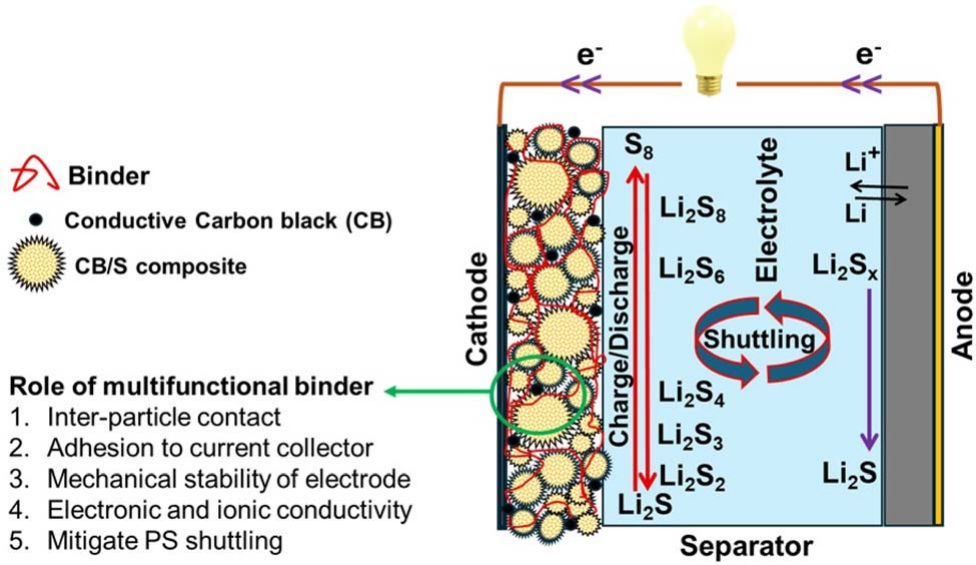
Schematic of the components, working mechanism and PS shuttling phenomenon of Li–S batteries during cycling. This figure also highlights the critical role of binders in achieving optimum performance and stability of Li–S cathodes.

The working principle of Li–S batteries is based on a multistep conversion reaction of elemental S at the cathode. At the cathode, elemental S_8_ undergoes stepwise reduction to form soluble long-chain lithium PS (Li_2_S_8_–Li_2_S_6_), which are further reduced to medium- and short-chain species (Li_2_S_4_–Li_2_S_2_), ultimately forming insoluble Li_2_S as the end-product of discharge. During charging, this process reverses, regenerating elemental S_8_, Fig. 1. Although this conversion reaction provides high theoretical specific capacity and energy density, the formation and dissolution of intermediate PS lead to challenges such as the shuttle effect, active material loss, significant cathode volume expansion and interfacial instability, all of which severely impact the electrochemical performance of Li–S batteries.^10,13,14^

## 3. Role of binders in delivering high-performance and stable Li–S cathodes

Although the binder is present in a small percentage (5–10%) in the Li–S cathode, it must serve multiple critical functions for optimal battery performance. The binder plays an important role in maintaining electrode integrity by binding the active material and conductive additives, while ensuring strong adhesion to the current collector with minimal interfacial resistance.^23,26^ In Li–S batteries, its function is more demanding than in conventional Li-ion systems. Unlike Li-ion batteries, where solid–solid reactions take place during battery cycling, Li–S batteries undergo solid–liquid–solid transitions.^27,28^ These phase transformations lead to significant volume changes in the cathode (80%) and expose the binder to repeated physical and chemical stress, directly affecting the structural stability, active material utilization and long-term performance.^29,30^

The binder in the Li–S cathode must provide mechanical stability and accommodate high volume changes during cycling, thereby preserving electrical connectivity and maintaining a strong interfacial contact. Additionally, binders should chemically interact with soluble PS species to mitigate PS shuttling. It should facilitate electron and ion conductivity within the electrode and support scalable processing (Fig. 1). Therefore, the binder in Li–S systems is not merely a passive adhesive, but a functional material whose mechanical, chemical and transport properties collectively govern the rate capability, active material utilization and cycling stability.

### 3.1. State-of-the-art polymer binders and challenges associated with their use in Li–S cathodes

Traditional polymer binders such as polyvinylidene fluoride (PVDF), polytetrafluoroethylene (PTFE) and other inert polymeric materials have been widely used in Li–S batteries due to their simple structure, chemical stability and established processing methods.

However, the complex electrochemical environment of Li–S systems imposes demands that far exceed the capabilities of these inert materials, leading to capacity fading and poor long-term stability.^31^

• Conventional binders are non-polar and exhibit little chemical affinity towards lithium PS, allowing soluble species to migrate into the electrolyte and accelerate the shuttle effect.^31^

• They are also mechanically rigid and unable to accommodate the large (∼80%) volume expansion of the cathode during cycling, resulting in electrode cracking, particle detachment and loss of electrical contact.^14,23,32^

• Most of the traditional polymer binders are electronically and ionically insulating. They do not contribute to the charge transport within the cathode and hinder electron and Li-ion movement, contributing to sluggish redox kinetics and poor rate capability.^33,34^

• Traditional binders lack the ability to regulate Li_2_S nucleation and growth, often resulting in the uneven deposition of thick insulating layers in the cathode. These deposits block the active sites, increase polarization and reduce the reversibility of S redox reactions during charging.^31,35^

• Long-term exposure to PS and electrolyte solvents can weaken the polymer chains, reduce their adhesion strength and alter their mechanical properties.^31^

• PVDF-based cathodes require toxic solvents such as NMP for processing, raising environmental and manufacturing concerns.^36^ These limitations of traditional binders like PVDF become more pronounced under practical conditions, including obtaining high S loading cathodes.^37,38^

**Fig. 2:**
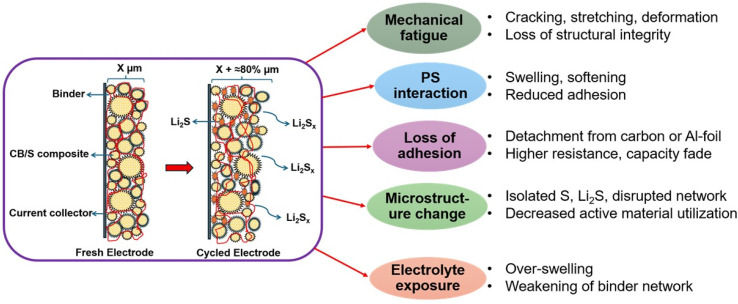
Schematic showing the S cathode degradation with cycling when conventional binder materials, such as PVDF and PTFE, are used. This figure highlights issues that occur in the presence of conventional binders.

Fig. 2 highlights the multiple degradation processes that take place in the S cathode when conventional polymer binders are used in Li–S batteries. Traditional binders are often unable to effectively accommodate volume changes occurring during cycling. These volume changes often result in electrode deformation and loss of structural integrity. Conventional binders do not have affinity towards the PS species, leading to increased shuttling. They provide limited or no contribution to electronic and ionic transport conductivity within the electrode. Electrolyte-induced swelling or electrode structural instability further affects the cathodes life. Overall, inadequate PS shuttling mitigation, poor accommodation of volumetric changes and insufficient charge-transfer result in rapid capacity fading and premature electrode failure. These limitations have resulted in the development of multifunctional binder systems that are specifically engineered to address the complex mechanical, chemical and electrochemical demands of high-performance Li–S cathodes.

### 3.2. Ideal properties of an Li–S cathode polymer binder

An ideal polymer binder for Li–S cathodes must meet several key requirements to stabilize the electrode during repeated cycling: (a) mechanical flexibility and strong adhesion to accommodate the large volume expansion during cycling;^39^ (b) maintain optimal interparticle contact between S, conductive additives and current collectors;^40^ (c) presence of polar or chemically active groups that interact with lithium PS to suppress their dissolution and migration, helping to mitigate the PS shuttle effect;^40,41^ (d) allow efficient ion and electron transport, either by facilitating lithium ion diffusion through the polymer matrix or by contributing to intrinsic conductivity;^42^ (e) chemical and electrochemical stability within the voltage window, minimal swelling in the electrolyte and compatibility with high S loading and lean electrolyte conditions;^40,42,43^ and (f) from a practical perspective, polymer binders should support scalability and be environmentally friendly. They should be cost-effective, highly reproducible and have long-term durability to enable commercial viability.^27,39^

## 4. New-generation polymer binders for Li–S cathodes

**Fig. 3:**
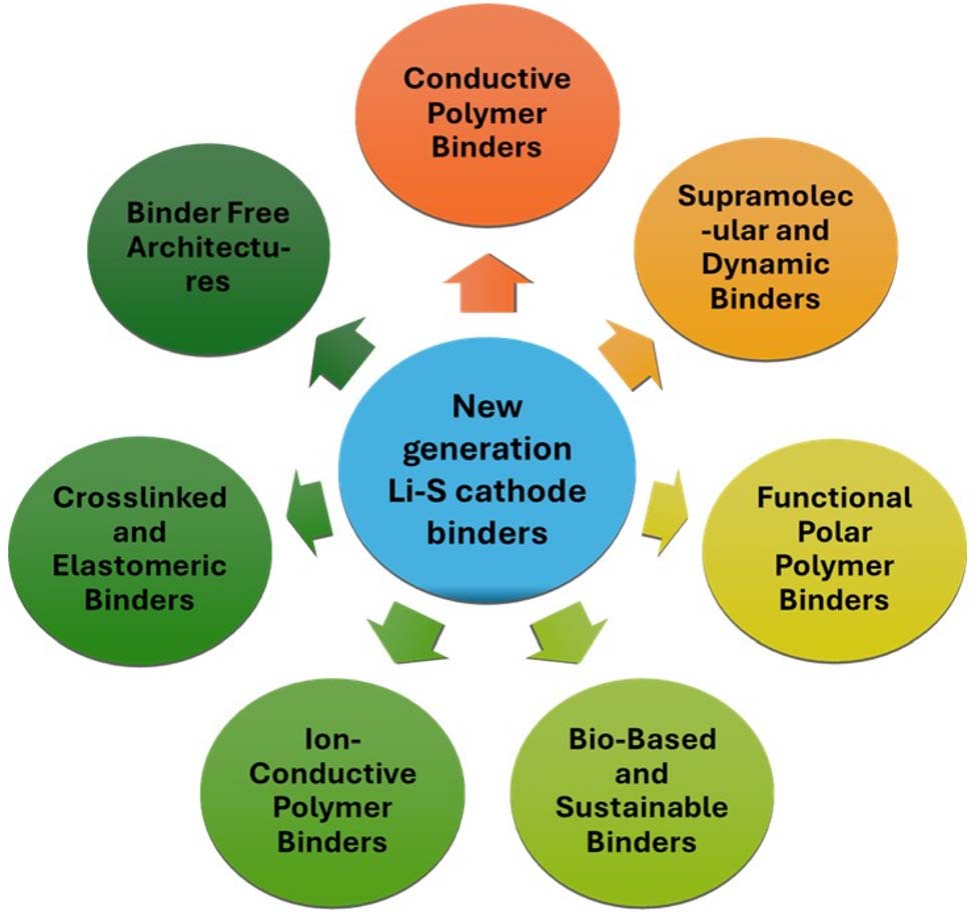
Classification of emerging binder systems for Li–S cathodes based on their additional functional roles beyond conventional mechanical binding. This figure categorizes binders according to their functionalities such as PS anchoring, ionic and electronic conductivity enhancement, electrocatalytic activity, self-healing capability, elastic volume-buffering, and sustainable/green processability, highlighting how these multifunctional designs address the intrinsic challenges of Li–S battery chemistry.

Recent advances in Li–S battery research have driven the development of new-generation cathode binders that extend beyond the simple mechanical adhesion of the electrode components. Fig. 3 shows the different classes of binders that have been used to improve the Li–S cathode optimization and battery performance.

### 4.1. Conductive polymer binders

**Fig. 4:**
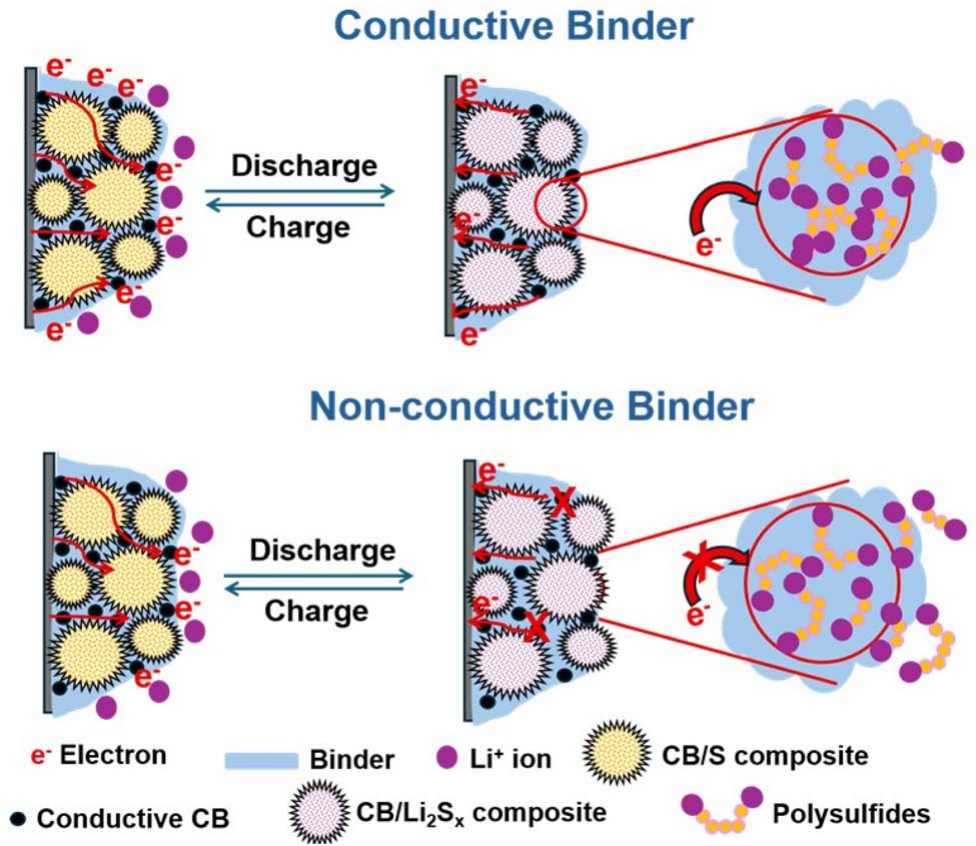
Schematic of the working mechanism of conductive and non-conductive binders in Li–S batteries.

Conductive polymer binders are a class of advanced materials for Li–S cathodes, which combine mechanical strength with intrinsic conductivity. Unlike conventional insulating binders, these binders facilitate charge transport, addressing the low electronic conductivity of S and its discharge products. In Li–S cathodes, conductive polymer binders create a conductive network linking active material particles and carbon additives, ensuring continuous electron flow during cycling. Their conjugated polymer backbones enable electron delocalization, while polar functional groups bind lithium PS through electrostatic attraction or hydrogen bonding, as represented in Fig. 4. This dual function constrains PS dissolution and prevents electronic isolation of the active material due to volume expansion and Li_2_S deposition. The conductive network boosts active material utilization, accelerates redox kinetics, and improves the rate capability and cycling stability.^42,44–46^ A few examples of conductive binders include poly(3,4-ethylenedioxythiophene): poly(styrene sulfonate) (PEDOT:PSS), polyaniline (PAN)-based binders and polypyrrole (PPy)-based binders.

**Fig. 5:**
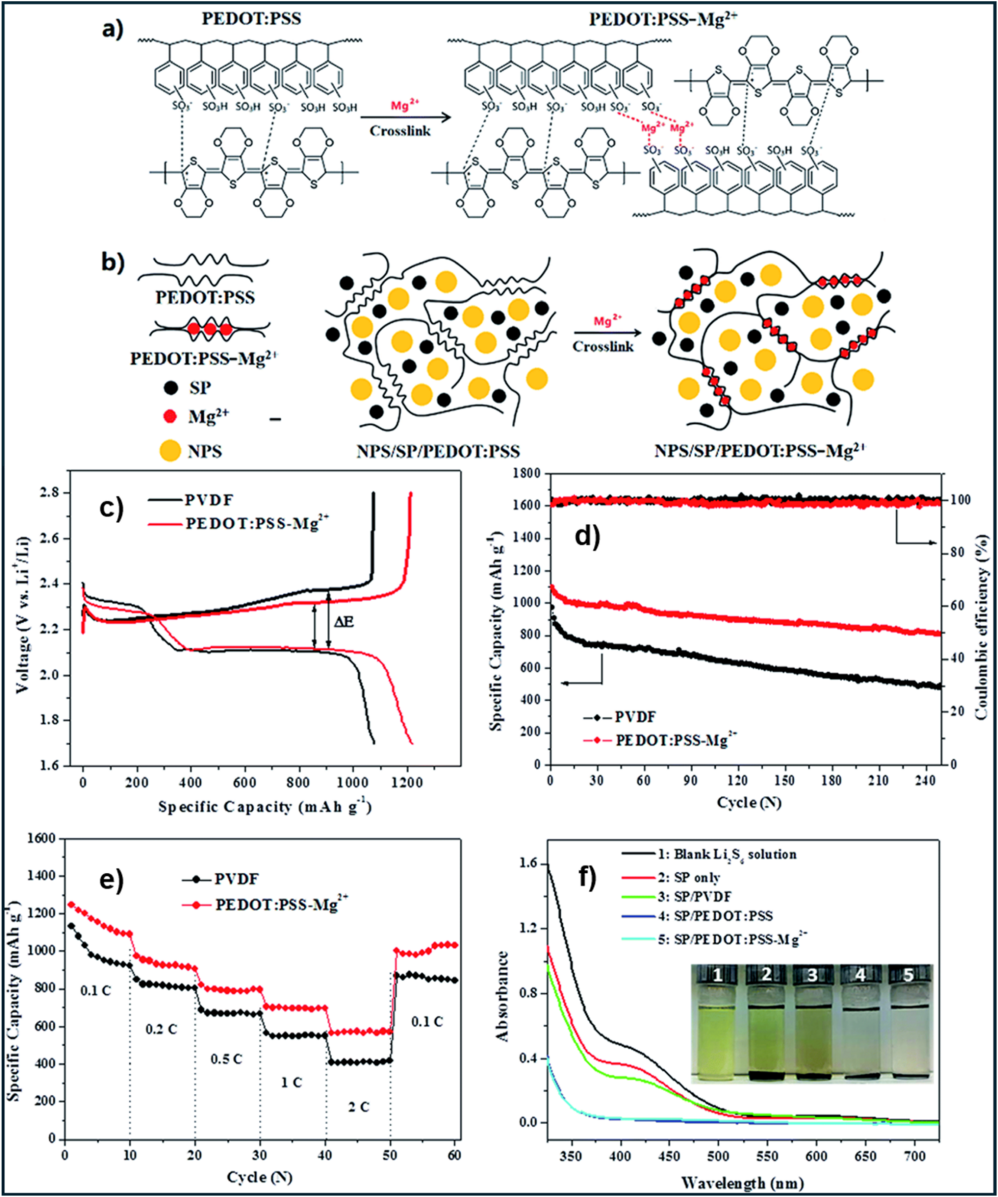
Schematic of the (a) PEDOT:PSS-Mg^2+^ binder structure and NPS/SP/PEDOT:PSS-Mg^2+^ electrode. (b) Coordination of Mg^2+^ by SO_3_^−^ is the main process in the gel formation and polymer-metal framework stabilization. Discharge–charge performance of the NPS/SP/PVDF and NPS/SP/PEDOT:PSS-Mg^2+^ electrodes: (c) typical discharge–charge voltage *vs.* capacity profile, (d) cycling performance at 0.5C, and (e) rate performance from 0.1C to 2C. (f) UV-vis absorption spectra and photographs of the Li_2_S_6_ solutions in 1,3-dioxolane (DOL)/1,2-dimethoxyethane (DME) (v/v, 1/1) before (blank) and after the addition of SP, SP/PVDF, SP/PEDOT:PSS and SP/PEDOT:PSS-Mg^2+^.^47^ Reproduced from ref. 47 with permission from the Royal Society of Chemistry.

PEDOT:PSS is one of the most extensively studied conductive polymer binders for Li–S cathodes because of its superior electrical conductivity, flexible characteristics and water processability. The conjugated backbone of PEDOT facilitates electrical conduction, whereas the sulfonate groups of PSS enable substantial interactions with PS. The sulfonic acid groups enhance PS anchoring and increase interfacial stability, thus mitigating the shuttle effect. Furthermore, PEDOT:PSS produces homogenous thin films that improve adhesion between active material particles, the carbon host and the current collector.^47–49^ Yan *et al.*^47^ reported a conductive multifunctional polymer binder that was based on Mg^2+^-crosslinked PEDOT:PSS. A binder was developed to simultaneously tackle several key challenges in Li–S batteries. The intrinsically conductive PEDOT:PSS framework enhanced the electron transport throughout the S cathode, improving the overall electrode conductivity. Meanwhile, coordination crosslinking with Mg^2+^ ions generated a mechanically robust three-dimensional network capable of accommodating the substantial volume fluctuations associated with S redox reactions, Fig. 5a and b. In addition, the oxygen-rich functional groups within the polymer matrix provided strong chemical interactions with dissolved PS, effectively mitigating shuttling and promoting reaction stability. The voltage/capacity profiles recorded during the second galvanostatic discharge/charge cycle revealed the electrochemical behavior of cells employing nano-particulate sulfur/Super P (NPS/SP) cathodes with different binders. The cathode incorporating the Mg^2+^-crosslinked PEDOT:PSS binder exhibited improved redox characteristics (≈1225 mAh g^−1^, lower voltage hysteresis) compared with the conventional PVDF-based counterpart. For the NPS/SP/PVDF electrode, a specific discharge capacity of 1079 mAh g^−1^ was achieved at a current rate of 0.1C, serving as a benchmark for evaluating the enhanced performance enabled by the multifunctional conductive binder, Fig. 5c. Owing to these synergistic effects, Li–S cells employing this binder delivered an initial discharge capacity as high as 1097 mAh g^−1^ and maintained 74% of their capacity after 250 cycles at 0.5C even with a high S loading of 70 wt% in the cathode, markedly outperforming conventional PVDF-based systems, Fig. 5d. C-rate measurements also indicated an improved performance by the PEDOT:PSS-based cathode compared with that by the PVDF-based cathode, Fig. 5e. PS confinement by PEDOT:PSS was further confirmed by UV-vis spectroscopy data, Fig. 5f. Beyond the electrochemical advantages, this binder also enabled water-based electrode processing, eliminating the need for toxic organic solvents such as NMP, thereby offering a more sustainable fabrication route.

**Fig. 6:**
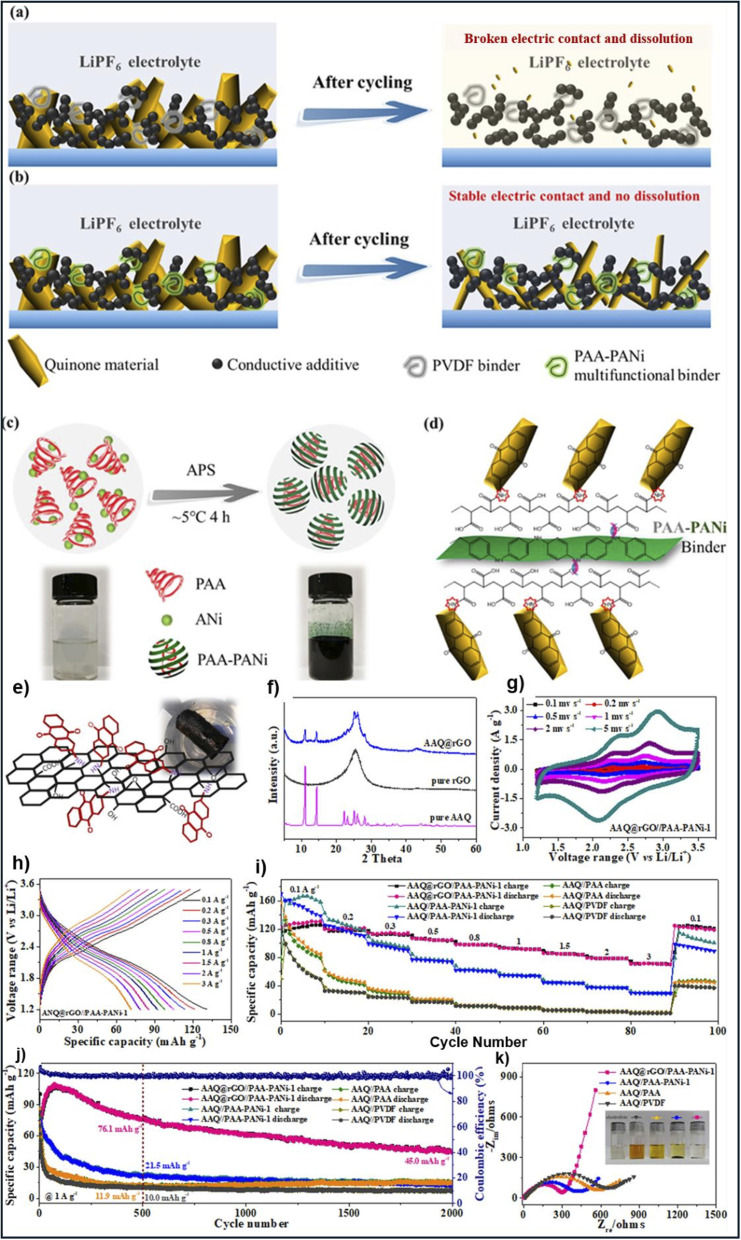
Comparison of the AAQ electrode with the (a) PVDF binder and (b) PAA-PANi multifunctional binder before and after cycling. (c) Schematic showing the synthetic process for the PAA-PANi multifunctional binder. (d) Chemical structure of the PAA-PANi binder and AAQ-based organic electrode materials and their chemical interaction. (e) Schematic of the interaction between AAQ and rGO; inset is a photograph of the AAQ@rGO xerogel. (f) XRD profiles of AAQ, rGO, and the AAQ@rGO composite. (g) CV curves and (h) GCD curves of the AAQ@rGO electrode prepared with the PAA-PANi-1 binder. (i) Specific capacities and (j) capacity retention of the AAQ electrodes prepared with the PVDF, PAA, and PAA-PANi-1 binders and the AAQ@rGO electrode prepared with the PAA-PANi-1 binder. (j) Specific capacity at a current density of 1 A g^−1^; the right-hand column is the corresponding coulombic efficiency. (k) EIS of AAQ electrodes prepared with PVDF, PAA, and PAA-PANi-1 binders and the AAQ@rGO electrode prepared with the PAA-PANi-1 binder. EIS was measured after 3 cycles of the activation process; inset is a photograph of the corresponding electrodes soaked in the electrolyte for 12 h.^53^ Copyright 2020 by American Chemical Society.

PAN has attracted considerable attention as a cathode material and electrolyte component in Li–S batteries.^50–52^ Sulfurized polyacrylonitrile (SPAN) has gained attention as an effective cathode material for addressing the challenges of conventional carbon/S composite electrodes. In SPAN, S is chemically confined within a carbon–nitrogen polymer framework through strong covalent interactions, which effectively mitigates PS dissolution and suppresses the shuttle effect, thereby enhancing cycling stability.^50,51^ PAN offers several attractive characteristics, including excellent thermal resistance, broad electrochemical stability and robust mechanical properties when used in solid-state electrolytes.^52^ PAN can also serve as a conductive binder in Li–S batteries owing to its intrinsic properties. Tong *et al.*^53^ showed one such notable example using a conductive interpenetrating polymer network formed through *in situ* polymerization of PAN within a poly(acrylic acid) (PAA) in a Li-ion battery. The developed multifunctional PAA-PANi-1 binder showed enhanced electronic pathways with a conductivity of ∼10^−3^ S cm^−1^, while the PAA component acts as a robust binding matrix that interacts with the amino (−NH_2_) groups of organic 2-aminoanthraquinone (AAQ), mitigating active material dissolution and preserving structural integrity during repeated cycling, Fig. 6a and b. The PAA-PANi composite binder is synthesized through *in situ* polymerization of aniline within a PAA matrix. Initially, aniline and H_2_SO_4_ are introduced into an aqueous PAA solution, followed by the addition of (NH_4_)_2_S_2_O_8_ to initiate polymerization and form conductive PANi chains. During this process, electrostatic interactions and hydrogen bonding between protonated PANi and the carboxyl groups of PAA generate an interpenetrating polymer network, imparting both enhanced conductivity and mechanical robustness, Fig. 6c and d. Following a hydrothermal process, a freestanding, conductive AAQ@rGO aerogel was formed through a one-step epoxy ring–opening reaction between amino groups in AAQ and epoxy groups in GO, Fig. 6e. XRD analysis confirmed the characteristic features of both AAQ and rGO, Fig. 6f. Notably, the AAQ@rGO electrode using the PAA-PANi binder retained stable CV profiles as the scan rate increased from 0.1 to 5 mV s^−1^, indicating minimal polarization and favorable reaction kinetics, Fig. 6g. Benefiting from the combined conductivity and strong mechanical adhesion of the binder, the AAQ/reduced graphene oxide (AAQ@rGO) electrode exhibited higher battery performance. The batteries showed a high specific capacity of 126.1 mAh g^−1^ at 0.1 A g^−1^ and 71.3 mAh g^−1^ at a high current density of 3 A g^−1^, and demonstrated remarkable long-term stability over 2000 cycles at 1 A g^−1^, Fig. 6h and i. Batteries with the PAA-PANi-1 binder also showed enhanced performance in rate capability tests and low resistances, as shown by impedance spectroscopy (EIS), Fig. 6j and k. These performances significantly surpassed those of conventional PVDF and PAA-PAN-based binder systems, underscoring the potential of conductive polymer binders as an effective approach to address poor conductivity and dissolution issues in organic electrode materials.^53^ This approach can significantly help in increasing the performance of Li–S batteries.

Other polymers such as PPy are promising conductive binders for Li–S cathodes. PPy has high electronic conductivity, chemical stability and is easy to synthesize. PPy contains heteroatoms that interact with PS, helping their encapsulation, while the conjugated system provides electronic conductivity. When used as a binder, PPy forms a conductive coating around the S particles, enhancing the electronic connectivity and preventing S dissolution. However, its rigid structure can limit its ability to accommodate large-volume expansions, necessitating composite designs or a flexible polymer blending to improve the mechanical durability.^54–57^Table 1 summarizes the advantages, limitations and future directions of conductive polymer binders.

**Table 1:** Summary of the advantages, limitations and future research directions of conductive binders^58–60^

Advantages	Limitations	Future research directions
• Provides continuous electronic pathways	• Higher cost compared to conventional binders	• Enhance cost-effectiveness
• Enhances S utilization	• Chemical degradation in PS-rich environments	• Employ strategies such as polymer blending and crosslinking
• Reduces reliance on excess conductive carbon additives	• Limited mechanical flexibility (for some polymers)	• Achieve synergistic improvements in conductivity, elasticity and PS confinement
• Improves the redox kinetics and rate performance	• Complex synthesis and processing requirements	• Enable high performance under practical Li–S battery conditions

### 4.2. Supramolecular and dynamic binders

**Fig. 7:**
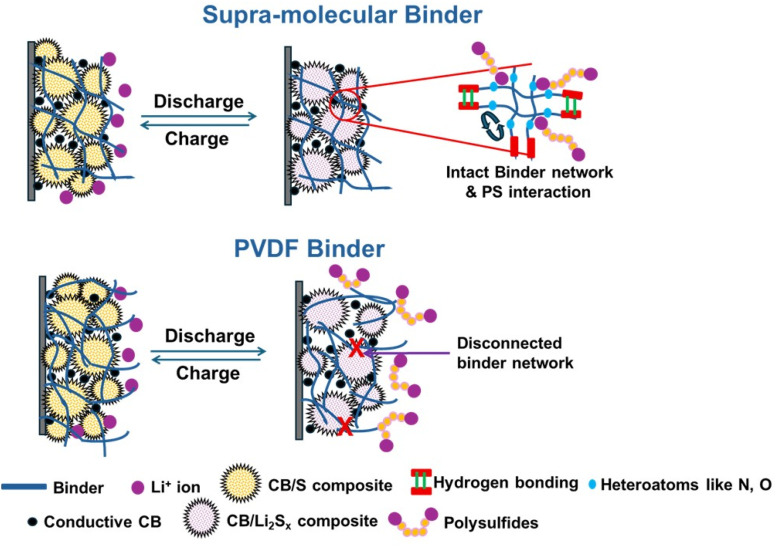
Schematic of the working mechanism of the supramolecular and PVDF binders during battery cycling.

Supramolecular and dynamic binders are considered as promising advanced binder materials for Li–S cathodes, offering adaptive mechanical and chemical functionalities that conventional polymer binders cannot provide. Unlike static binders that rely on permanent covalent bonds, supramolecular and dynamic binders incorporate reversible interactions. This includes hydrogen bonding, metal–ligand coordination, imine bonding, borate ester linkages or ionic interactions, which can break and reform during electrochemical cycling.^41,61,62^ The dynamic nature allows the binder network to self-adjust in response to the severe mechanical and chemical stresses known in Li–S batteries. During repeated cycling, S undergoes large volumetric changes and generates soluble PS that destabilize the cathode, Fig. 7. Supramolecular binders accommodate these changes through reversible bonding, which enables stress dissipation and self-healing. When mechanical strain is induced by S expansion or contraction, dynamic bonds temporarily dissociate, allowing polymer chains to rearrange and relieve stress, and then reassemble to restore the structural integrity.^41,63,64^

Polymer binders abundant in donor and acceptor hydrogen bonds, such as ureidopyrimidinone (UPy)-based polymers, polyacrylic acid-based networks and hydroxyl-rich biopolymers, establish vast hydrogen-bonded networks. These interactions offer robust yet reversible cohesion, facilitating effective accommodation of volume expansion while preserving close contact between S, carbon and conductive additives. Hydrogen bonding promotes PS adsorption, hence improving the cycling stability.^65–67^

**Fig. 8:**
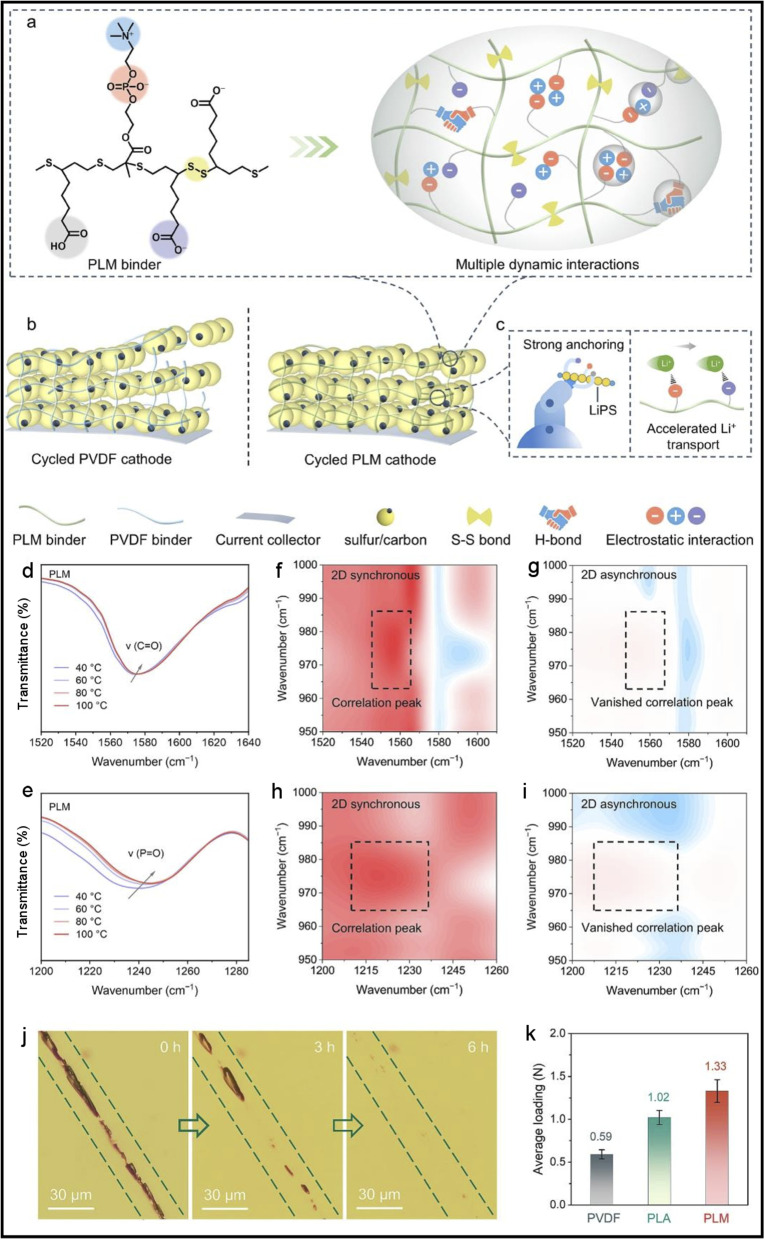
Schematic of the structure and function of the PLM binder. (a) Typical functional structures and dynamic interactions within PLM. (b) Unstable cycled PVDF cathode. (c) Intact cycled PLM cathode and multiple functions of the PLM binder. Dynamic interactions and self-healing properties. Temperature-dependent FTIR spectra of PLM at the regions of (d) *v*(C=O), belonging to –COO^−^, and (e) *v*(P=O). (f and g) 2DCOS synchronous and asynchronous spectra corresponding to (d), respectively. (h and i) 2DCOS synchronous and asynchronous spectra corresponding to (e), respectively. (j) Scratch test of the PLM binder. (k) Average peeling force of the PVDF, PLA, and PLM cathodes.^64^ Copyright 2020 by Elsevier.

Lin *et al.*^64^ reported a multi-functional zwitterionic polymer binder (PLM) to address the key limitations in Li–S batteries through synergistic dynamic interactions, including electrostatic attraction, hydrogen bonding and reversible disulfide linkages. These interactions created a self-healing cross-linked network that improved mechanical resilience and stabilized the S cathode during cycling. The binder's abundant polar functionalities strongly adsorbed PS, suppressing shuttle effects, while phosphate and carboxylate anionic groups promoted Li^+^ transport and accelerated ion diffusion, Fig. 8a–c. Temperature-dependent FTIR (40–110 °C) revealed dynamic chemical interactions within the PLM binder, in which the C=O in –COO^−^, P=O and –N^+^(CH_3_)_3_ peaks shifted to higher wavenumbers with reduced intensity, indicating thermally responsive bonding behavior, Fig. 8d and e. Two-dimensional correlation FTIR further confirmed the electrostatic interactions between carboxylate/phosphate anions and quaternary ammonium cations, along with hydrogen-bonding interactions involving –COOH groups, while reversible S–S bonds contributed additional dynamic crosslinking. Together, these interactions establish a robust adaptive network with intrinsic self-healing capability, Fig. 8f–i. Scratch-healing tests showed substantial recovery within 3 h and nearly completed healing after 6 h, accompanied by a high healing efficiency of 94.32%, Fig. 8j. Beyond self-healing, PLM also showed strong adhesion in 180° peel tests. Compared to other binders, PLM showed the highest peeling force, indicating structural stability during extended cycling, Fig. 8k.

**Fig. 9:**
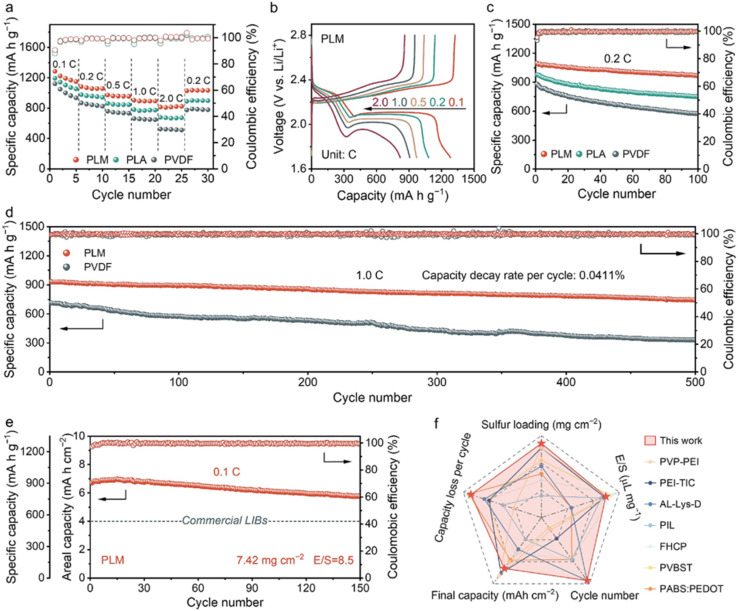
Electrochemical performance. (a) Rate performance. (b) Charge–discharge curves of the PLM cathode. Long-term cycling performance at (c) 0.2C and (d) 1.0C. (e) Cycling performance of the PLM cathode with a S loading of 7.42 mg cm^−2^. (f) Comparison of several recent binder-related works in electrochemical performance under high S loadings.^64^ Copyright 2020 by Elsevier.

PLM-based cathode batteries showed superior battery performance in rate capability studies. At 0.2C, the PLM cathode delivered a high initial capacity of 1096.3 mAh g^−1^ and retained 88.3% capacity after 100 cycles, outperforming PLA and PVDF cathodes, which showed lower capacity retentions of 76.6% and 65.2%, respectively, Fig. 9a–c. Benefiting from these combined effects, Li–S cells with the PLM binder exhibited outstanding durability, with an ultralow capacity fading rate of 0.041% per cycle over 500 cycles at 1.0C, Fig. 9d. Under practical conditions, the electrode maintained a high areal capacity of 5.46 mAh cm^−2^ after 150 cycles at a S loading of 7.42 mg cm^−2^. Even at a higher S loading of 11.45 mg cm^−2^ with a lean electrolyte amount of 6.4 µL mg^−1^, it delivered 7.64 mAh cm^−2^ after 80 cycles, underscoring the promise of PLM binders for high-performance Li–S batteries, Fig. 9e. As shown in Fig. 9f, excellent cycling stability was achieved even at high S loadings by the PLM-based cathode compared to other binders.

A few other examples (including the silicon-based anodes for Li-ion batteries) use dynamic covalent binders, where moieties with reversible covalent connections, such as imines (Schiff bases), borate esters or disulfide links, are present and can be considered for S cathodes. These bonds provide superior strength compared to non-covalent contacts while maintaining reversibility in electrochemical environments. Imine-based binders demonstrate self-healing properties and mechanical strength, significantly mitigating the electrode fracture. Borate ester networks provide flexibility and ion-conductive routes, enhancing lithium-ion transport in the cathode.^68,69^ Supramolecular binders with metal–ligand coordination have also shown strong yet reversible interactions between the polymer ligands and metal ions (*e.g.*, Al^3+^). These coordination bonds enhance the mechanical strength and provide additional Lewis acid-base sites for PS interactions. They also exhibit improved resistance to mechanical fatigue and superior PS confinement during long-term cycling.^70,71^Table 2 summarizes the advantages, limitations and future directions of supramolecular and dynamic binders.

**Table 2:** Summary of the advantages, limitations and future research directions of supramolecular and dynamic binders^34,72,73^

Advantages	Limitations	Future research directions
• Self-healing capability under mechanical stress	• Complex synthesis routes	• Integration of multifunctional properties (self-healing + conductivity)
• Effective accommodation of large volume changes	• Sensitivity of dynamic bonds to electrolyte composition	• Development of binders with both electronic and ionic conductivity
• Enhances PS confinement and reduced shuttle effect	• Trade-off between the mechanical strength and reversibility	• Scalable and cost-effective fabrication strategies
• Improves the electrode integrity and cycling durability	• Provides no conductivity	• Design of robust binders for practical, high-performance Li–S cathodes
• Suitable for high S loading electrodes		

### 4.3. Functional polar polymer binders

**Fig. 10:**
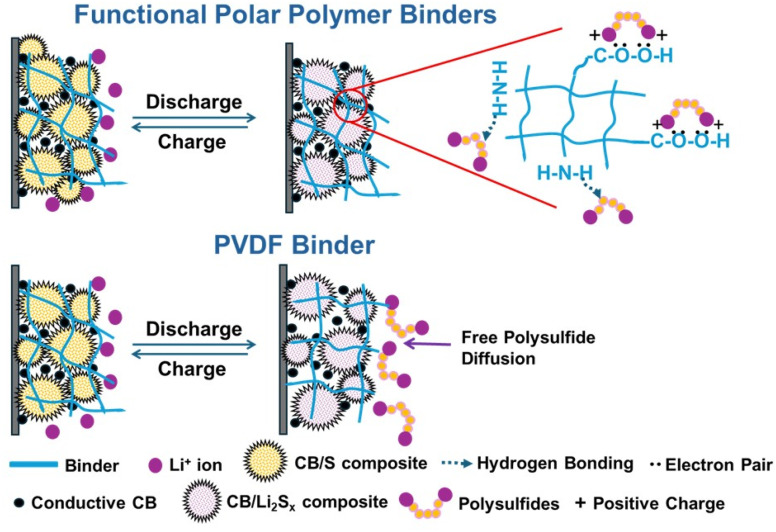
Schematic of the working mechanisms of a functional polar polymer binder compared with that of a PVDF binder during battery cycling.

Functional polar polymer binders have emerged as one of the most effective strategies for addressing the intrinsic challenges of Li–S cathodes. Unlike conventional inert binders, these materials are chemically active and contain polar functional groups such as carboxyl (–COOH), hydroxyl (–OH), amine (–NH_2_), sulfonate (–SO_3_H) and ether (–C–O–C–) that interact strongly with PS intermediates.^73,74^ By combining mechanical binding with chemical confinement, functional polar polymer binders play an important role in stabilizing the cathode structure, suppressing the PS dissolution and enhancing the long-term electrochemical performance. Functional polar polymer binders mitigate shuttling issues by forming strong interactions with the PS species through hydrogen bonding, electrostatic attraction and Lewis acid-base interactions, Fig. 10.^75^ Functional groups serve as PS anchoring sites that immobilize PS within the cathode region. In addition to chemical confinement, these binders maintain mechanical cohesion among S, conductive additives and the current collector during repeated volume expansion and contraction. Few examples include PAA, carboxymethyl cellulose (CMC) and polyethyleneimine (PEI).^74–77^

Carboxyl-containing polymer binders are molecules containing carboxyl groups, such as PAA, CMC and alginate. These are among the most extensively studied functional binders for Li–S cathodes. The –COOH groups provide strong electrostatic interactions with PS, effectively reducing their solubility and diffusion. These polymers also exhibit excellent adhesion and flexibility, enabling them to accommodate large volume expansion. Moreover, their water solubility allows for environmentally friendly and scalable electrode fabrication.^74–78^ Amine-rich polymers, including PEI and crosslinked chitosan sulfate, offer strong chemical affinity towards PS through Lewis acid-base interactions between the nitrogen atoms and S species. These interactions significantly suppress the shuttle effect and enhance the capacity retention. Amine-containing binders also improve cathode wettability and interfacial stability, although excessive PS adsorption may sometimes slow down the redox kinetics if not carefully optimized.^79–81^

**Fig. 11:**
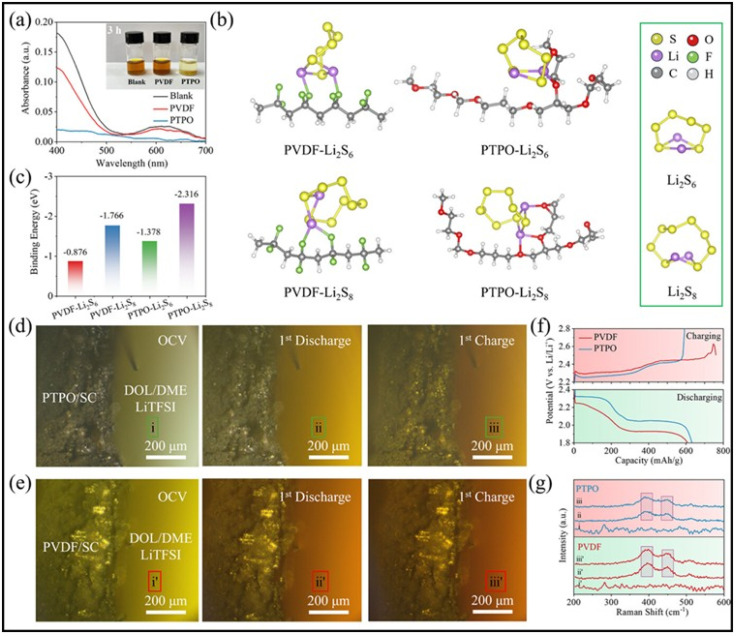
PS adsorption behavior and *in situ* electrochemical analysis of the PVDF and PTPO binders. (a) UV-vis absorption spectra of the blank solvent, PVDF and PTPO solutions. The inset displays the corresponding optical images from the LiPS adsorption tests. (b) Optimized molecular configurations of PVDF and PTPO interacting with representative LiPS species, as obtained *via* DFT calculations. (c) Calculated binding energies between the PVDF/PTPO binders and various LiPS species. (d and e) *In situ* optical microscopy images of the PTPO@S and PVDF@S systems during electrochemical cycling. (f) Galvanostatic charge–discharge profiles and (g) Raman spectra of the electrolytes from PTPO- and PVDF-based cells.^83^ Copyright 2025 by Wiley.

Sulfonated polymers and ether-containing polymers introduce highly polar functional groups that improve the electrolyte affinity and lithium-ion transport. Sulfonate groups provide strong electrostatic interactions with PS, while ether linkages facilitate lithium-ion coordination and transport within the binder matrix. These features enhance both chemical confinement and reaction kinetics, making such binders particularly attractive for high active material loading cathodes.^52,82,83^ Zhang *et al.*^83^ reported an epoxy-ether-based binder that can empower ultra-high S loading cathodes for Li–S batteries. However, realizing high-energy-density Li–S batteries under practical conditions, such as high S loading (≥5 mg cm^−2^) and lean electrolyte (E/S < 5 µL mL^−1^), remains a challenge due to the large volume fluctuations, unstable interfaces and severe PS shuttling. To address these issues, a 3D cross-linked polyether binder (PTPO) was developed through cationic copolymerization of glycerol triglycidyl ether (TEP) and 1,3-dioxolane (DOL). Owing to its flexible polymer network, strong interfacial adhesion and abundant ether/epoxy functionalities, PTPO accommodates cathode volume changes while chemically confining PS, thereby improving the structural and electrochemical stability. PTPO exhibits strong chemical affinity toward lithium PS, as evidenced by the near-baseline UV-vis absorbance after Li_2_S_6_ exposure in contrast to the pronounced absorption retained with PVDF, confirming its superior PS adsorption, Fig. 11a. DFT calculations revealed strong interactions between PTPO and PS, where Li_2_S_6_ and Li_2_S_8_ preferentially coordinate with the binder's ether and epoxy functionalities, Fig. 11b. The high binding energies of −1.77 eV for Li_2_S_6_ and −2.32 eV for Li_2_S_8_, significantly exceeding those of PVDF, confirm the superior PS affinity of PTPO, Fig. 11c. Due to strong chemical interactions, PS dissolution and shuttle effects are effectively suppressed, contributing to improved cycling stability. *In situ* optical visualization and Raman analysis confirmed these findings, showing minimal electrolyte discoloration and reduced dissolved PS for PTPO electrodes, in stark contrast to severe PS diffusion in PVDF systems. Together, these results highlight the strong PS immobilization capability of PTPO, crucial for mitigating the shuttle effects and enhancing the cycling stability, Fig. 11d–g.

**Fig. 12:**
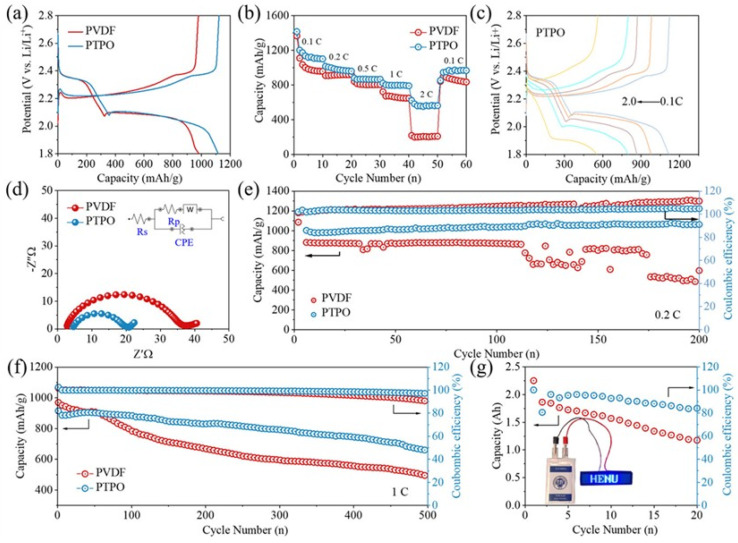
Electrochemical performance of Li–S batteries employing the PTPO and PVDF binders. (a) Galvanostatic charge–discharge profiles of Li–S cells utilizing the PVDF and PTPO binders at a current density of 0.1C. (b) Rate performance at a S loading of 3.5 mg cm^−2^ (1C = 1675 mAh g^−1^). (c) Galvanostatic charge–discharge profiles of PTPO-based Li–S cells under varying current densities. (d) Electrochemical impedance spectra (EIS) of Li–S cells employing the PVDF and PTPO binders after discharge, highlighting interfacial resistance differences. (e and f) Cycling stability of PTPO-based electrodes systematically evaluated at S loadings of ≈3.5 and ≈1.5 mg cm^−2^, corresponding to testing rates of 0.2C and 1C, respectively. (g) Long-term cycling performance of Li–S pouch cells assembled with PTPO-based cathodes.^83^ Copyright 2025 by Wiley.

Benefiting from these features, Li–S cells with the PTPO binder showed typical Li–S battery voltage curves and achieved better rate capability battery performance with increasing hysteresis at higher rates, Fig. 12a–c. Batteries with PTFO-based binders showed lower resistance compared to PVDF-based electrodes, Fig. 12d. The ether-rich PTPO framework enhances Li^+^ transport, lowers interfacial resistance and promotes faster redox kinetics. As a result, the PTPO cathode delivered stable cycling at 0.2C for 200 cycles, Fig. 12e. At 1C, an initial capacity of 915.2 mAh g^−1^ was obtained, and after 500 cycles, 658.1 mAh g^−1^ was retained, which corresponds to 99.93% capacity retention, thus significantly outperforming the PVDF cathode, Fig. 12f. Fig. 12g shows the Li–S pouch cell successfully powering an LED array, demonstrating its practical application potential.^83^

In addition to the chemical functionality, functional polar polymer binders exhibit improved mechanical adaptability compared to traditional binders. Their flexible polymer backbones and strong interfacial adhesion help distribute mechanical stress during cycling. This prevents electrode cracking, particle detachment and delamination. Crosslinking strategies are often employed to further enhance the mechanical strength without sacrificing the flexibility.^34,39,84^Table 3 summarizes the advantages, limitations and future directions of functional polymer binders.

**Table 3:** Summary of the advantages, limitations and future research directions of functional polar polymer binders^77,85,86^

Advantages	Limitations	Future research directions
• Strong chemical anchoring of lithium PS	• Excessive electrolyte uptake and swelling	• Molecular-level design of binders
• Reduced shuttle effect and improved coulombic efficiency	• Potential weakening of mechanical integrity	• Optimization of functional group density and spatial distribution
• Enhanced mechanical stability under large volume changes	• Overly strong PS binding may slow redox kinetics and increase polarization	• Tailoring polymer architecture for improved performance
• Improved S utilization and cycling performance	• Chemical affinity, mechanical properties and transport behavior need balancing	• Hybrid systems combining polar binders with conductive or dynamic networks
• High active material loading		

### 4.4. Bio-based and sustainable binders

Bio-based and sustainable binders have gained increasing attention in Li–S battery research as environmentally friendly alternatives to conventional petroleum-derived polymer binders. Renewable natural materials offer advantages like low toxicity, biodegradability, cost-effectiveness and compatibility with water-based processing.^40,87–90^ Many bio-based polymers are characterized by the presence of numerous polar functional groups, which facilitate strong chemical interactions with lithium PS, thereby addressing significant challenges associated with Li–S cathodes.^88,89^ During cycling, soluble lithium PS migrate into the electrolyte, resulting in the loss of the active material. Bio-based binders address this issue through various synergistic mechanisms. Functional groups such as –OH, –NH_2_, –C–O–C– and –COOH moieties present in natural polymers interact with the PS species *via* hydrogen bonding and electrostatic attraction. These interactions effectively immobilize them within the cathode. Concurrently, the flexible polymeric framework accommodates the substantial volume expansion of S during lithiation. This preserves electrode integrity and interfacial contact.^40,89^ Commonly reported bio-based binders include polysaccharide-based binders, chitosan and nitrogen-containing biopolymers as well as protein and lignin-based binders.^40,87–90^

Polysaccharides are the most studied class of bio-based binders for Li–S batteries. Compounds like sodium alginate, CMC, cellulose nanofibers and starch derivatives are abundant in oxygen-containing functional groups that strongly interact with PS. Sodium alginate is particularly noted for its exceptional mechanical strength and elasticity, which are due to its ionic crosslinking capability, allowing for effective stress relaxation during cycling. These binders have shown enhanced capacity retention and reduced PS shuttling compared to traditional PVDF binders.^31,89^

**Fig. 13:**
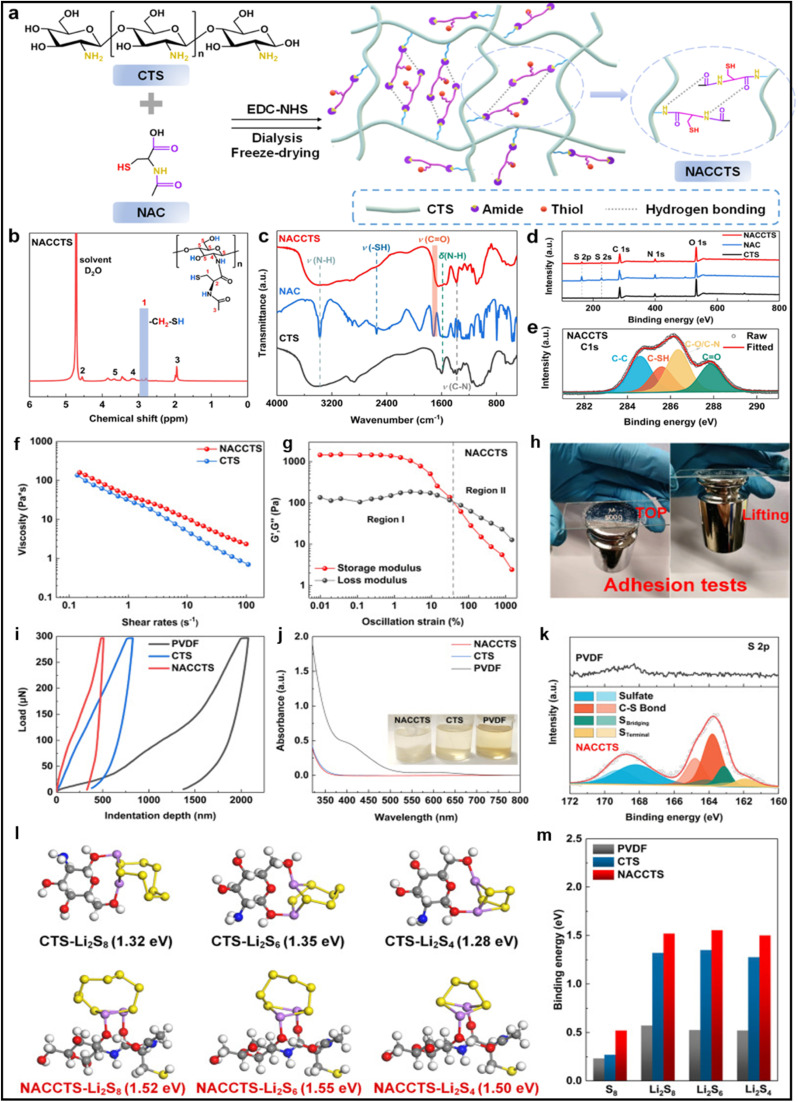
(a) Schematic of the synthesis process and chemical structure of the NACCTS binder. (b) ^1^H NMR spectra of NACCTS. (c) FTIR spectra of CTS, NAC and NACCTS. (d) Total XPS spectra of CTS, NAC, NACCTS and (e) C 1s XPS spectrum of NACCTS. (f) Shear rheological properties of the CTS and NACCTS electrode slurries. (g) Oscillation rheological properties of the CTS and NACCTS electrode slurries. (h) Adhesion property tests of the NACCTS binder. (i) Nanoindentation testing of PVDF-, CTS- and NACCTS-based cathodes. (j) UV-vis spectra of the sample solution after adsorption experiments with three binders. The inset is the optical photograph of the products after adsorption experiments. (k) S 2p XPS spectra of the PVDF and NACCTS binders after static adsorption. (l) Optimized chemisorption configurations and corresponding adsorption energies of Li_2_S_8_, Li_2_S_6_ and Li_2_S_4_ binding to CTS and NACCTS (gray balls represent carbon atoms, white balls represent hydrogen atoms, red balls represent oxygen atoms, blue balls represent nitrogen atoms, purple balls represent lithium atoms and yellow balls represent S atoms). (m) Adsorption energy of PVDF, CTS and NACCTS with S_8_ and Li_2_S_*x*_ (*x* = 4, 6, and 8).^88^ Copyright 2024 by Elsevier.

Jiang *et al.*^88^ introduced an aqueous, biomaterial derived polymer binder that was developed *via* a simple amidation strategy. The synthesized binder system enabled synergistic adsorption and conversion of PSs. The *N*-acetyl-l-cysteine-chitosan (NACCTS) binder was synthesized *via* the 1-(3-dimethylaminopropyl)-3-ethylcarbodiimide methiodide-*N*-hydroxysuccinimide (EDC-NHS)-mediated amidation between *N*-acetyl-l-cysteine (NAC) and chitosan (CTS) at room temperature, forming a hydrophilic polymer rich in amide–thiol–amide structures and hydroxyl groups. The amide groups enabled strong chemisorption of PS, while thiol groups form reversible S–S bonds, promoting redox-mediated conversion and stabilizing S species. This adsorption-conversion synergy enhanced reaction kinetics, while extensive hydrogen bonding from amide and hydroxyl groups improved the adhesion and mechanical strength, Fig. 13a. Structural characterization confirms successful synthesis, ^1^H NMR showed characteristic NAC signals at 2.7–2.9 ppm (thiol-adjacent methylene), 4.5 ppm and 2 ppm, Fig. 13b. FTIR reveals amide C=O stretching at 1660 cm^−1^ and N–H vibrations at 3370 and 1585 cm^−1^, indicating complete amidation, Fig. 13c. XPS detects S 2p and S 2s peaks at 163.8 and 228.6 eV, respectively, confirmed thiol incorporation. The C 1s spectrum further identified C–C (284.4 eV), C–S (285.4 eV), C–O/C–N (286.2 eV) and N–C=O (287.7 eV) bonds, verifying the chemical structure of NACCTS, Fig. 13d and e. Mechanical and rheological evaluations confirmed the superior structural performance of the NACCTS binder. Both CTS and NACCTS slurries exhibit shear-thinning behavior, ensuring good processability, while NACCTS showed higher viscosity due to stronger interactions from abundant oxygen-containing groups, Fig. 13f. Oscillatory rheology revealed a higher storage modulus and extended elastic-dominant region for NACCTS, indicating a stronger and more resilient network, Fig. 13g. Adhesion tests demonstrated robust bonding with NACCTS exhibiting nearly twice the peel strength of CTS and five times that of PVDF, and the ability to support a 500 g load. It also showed low swelling (13.78% *vs.* 62.48% for PVDF) and higher carbon residue in TGA, contributing to the structural stability and flame retardancy. Nanoindentation further confirmed the superior mechanical strength even under electrolyte-wetted conditions, Fig. 13h and i. In addition to mechanical robustness, NACCTS exhibits strong PS affinity. Fig. 13j shows that adsorption tests using 1 mM Li_2_S_6_ show near-complete decolorization for NACCTS and CTS, consistent with the disappearance of the 420 nm UV-vis peak. XPS analysis after adsorption identifies characteristic S species, including sulfate (169.2, 168.2 eV), C–S (164.8, 163.8 eV), bridging S (164.2, 163.2 eV) and terminal S (162.9, 161.9 eV), confirming strong chemical binding, whereas PVDF showed negligible interaction, Fig. 13k. Fig. 13l shows the optimized binding configurations and adsorption energies of Li_2_S_8_, Li_2_S_6_ and Li_2_S_4_ on CTS and NACCTS, highlighting atomic compositions (C: gray, H: white, O: red, N: blue, Li: purple, S: yellow). Additionally, comparative adsorption energies for PVDF, CTS and NACCTS with S species (S_8_ to Li_2_S) demonstrated a stronger binding affinity of NACCTS. These results highlight NACCTS as a mechanically robust and chemically active binder for stabilizing Li–S cathodes. Li–S cells using the *N*-acetyl-l-cysteine-chitosan (NACCTS) binder delivered a high initial discharge capacity of 1260.1 mAh g^−1^ at 0.2C, and exhibited excellent durability with a low-capacity fading rate of 0.018% per cycle over 400 cycles. Even at a high S loading of 8.4 mg cm^−2^, the system maintained strong areal capacity with 83.6% retention after 80 cycles. This approach highlights a sustainable, water-processable binder design that integrates chemisorption and redox mediation for practical high-performance Li–S batteries.^88^

Protein-based binders, such as gelatin and soy protein, offer multifunctional binding through a wide range of functional groups. These materials form robust adhesive networks and can accommodate electrode deformation during cycling. Lignin-based binders derived from biomass waste contain aromatic and polar functional groups that contribute to both mechanical reinforcement and PS confinement. Their rigid aromatic structure also improves thermal stability, making them promising candidates for sustainable electrode fabrication.^87,91,92^Table 4 summarizes the advantages, limitations and future directions of bio-based binders.

**Table 4:** Summary of the advantages, limitations and future research directions of bio-based binders^27,31,87^

Advantages	Limitations	Future research directions
• Derived from renewable and environmentally friendly sources	• Batch-to-batch variability	• Molecular engineering to enhance stability and conductivity
• Excellent mechanical flexibility and adhesion	• Limited electronic conductivity	• Improved interaction with PS
• Compatible with aqueous processing	• Potential swelling in liquid electrolytes	• Structural modification and crosslinking strategies
• Low-cost and scalable manufacturing	• Inferior long-term chemical stability in PS-rich environments	• Hybrid systems with conductive or synthetic polymers
• Reduced environmental impact	• Often requires modification or blending with other polymers	
	• Cannot be processed with Li_2_S in the presence of water solvent	

### 4.5. Ion-conductive polymer binders

Ion-conductive polymer binders have emerged as an advanced class of binders designed to provide mechanical integrity and facilitate lithium-ion transport within Li–S cathodes. Unlike conventional binders that are electrochemically and ionically inert, ion-conductive binders actively participate in ion transport processes. This participation reduces ionic resistance and improves S redox kinetics. Their multifunctional role makes them particularly attractive for high active material loading and thick electrodes. Where ion transport limitations become increasingly difficult in thick electrodes, lithium ions must diffuse efficiently through the cathode matrix to sustain the multi-step conversion reactions of S.^93,94^ Ion-conductive polymer binders contain chemical groups such as ether oxygen, carbonyl or sulfonate units that can temporarily bind lithium ions. By repeatedly attaching and releasing lithium ions, these groups allow the ions to move along the polymer chains. This process creates continuous pathways for lithium ion transport throughout the electrode, improving ion movement during battery operation. At the same time, the polymer network binds S and conductive additives, preserving the electrode cohesion during the large volume changes associated with S lithiation and de-lithiation. Poly(ethylene oxide) (PEO), lithium salt-containing polymer binders, gel-type and polymer electrolyte binders are among the few that have shown promising results.^93–97^

Nafion is one of the most studied ion-conductive polymer binders due to its strong affinity towards lithium ions and flexible molecular structure. The oxygen atoms in Nafion chains coordinate with lithium ions, enabling efficient ion transport through the cathode. When used as a binder in Li–S cathodes, Nafion improves ionic conductivity within the electrode and enhances S utilization. Incorporating lithium salts directly into polymer binders can enhance ionic conductivity by increasing the concentration of mobile charge carriers. Nafion is also known to be highly ion-selective as a result it allows only lithium-ions to pass through while blocking the migration of PS leading to reduced capacity fading and improved rate performance in Li–S cells.^98–101^

**Fig. 14:**
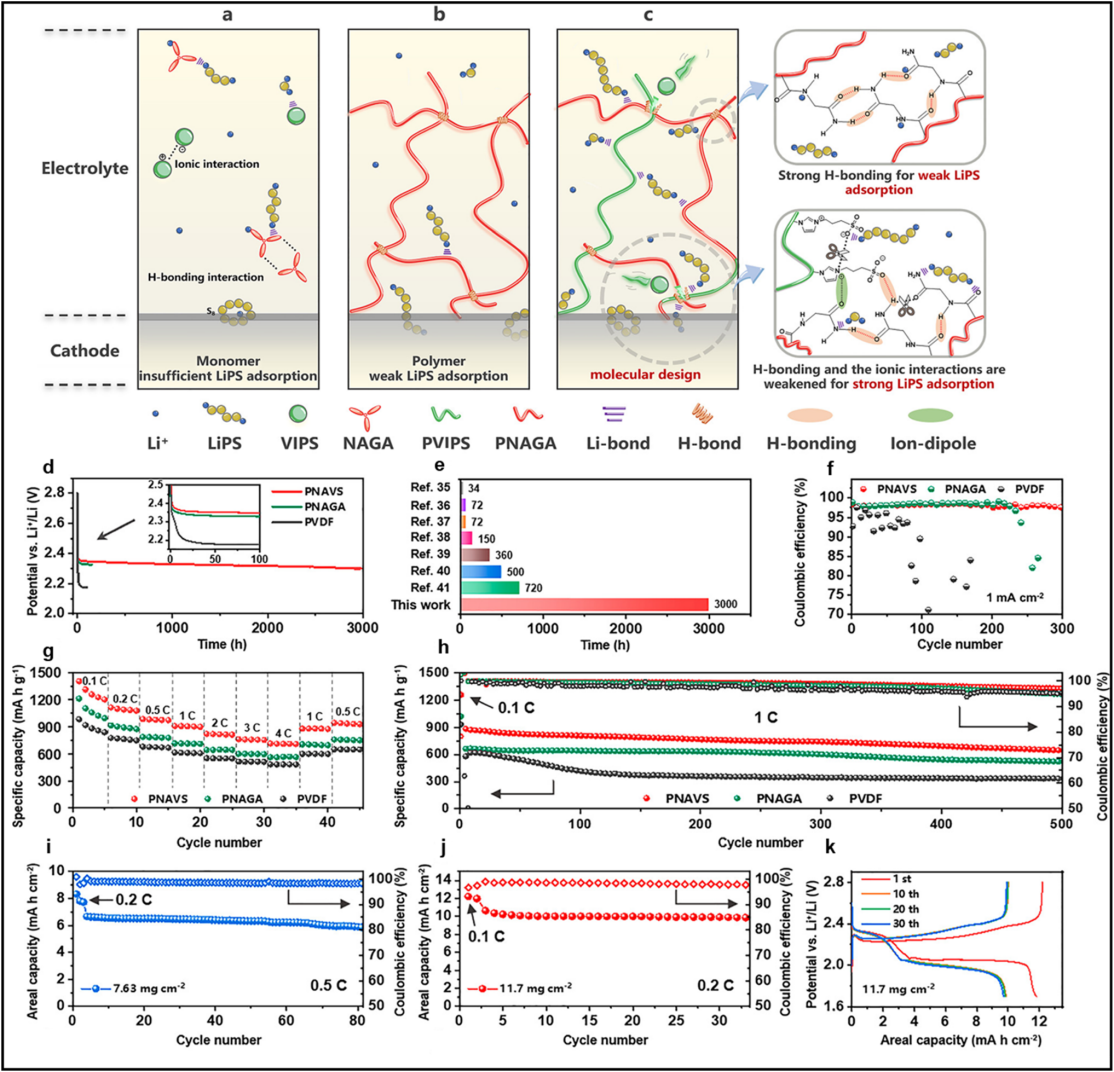
Schematic of the molecular interaction regulation: weakening the hydrogen bonding and ionic interactions in the binder for PS adsorption and redox. (a) NAGA monomers show insufficient interaction with lithium polysulfides (LiPSs), while the VIPS monomers can barely capture LiPSs due to the strong ionic interactions of the functional groups in VIPS. (b) PNAGA binder possesses weak LiPS adsorption due to the strong interchain hydrogen bonding. (c) After copolymerization, the PNAVS binder can effectively adsorb the LiPSs for shuttle effect inhibition and redox kinetics promotion. Electrochemical characterization and practical applications. (d) Self-discharge curves of the batteries with different binders and (e) comparison of the open circuit voltage (OCV) with other Li–S batteries. (f) coulombic efficiency of the asymmetrical Li/Cu cells with different binders. (g) Rate performance and (h) long-term cycling performance of Li–S batteries with different binders. Cycle performances of the battery with a PNAVS binder of high S loading for (i) 7.63 mg cm^−2^ at 0.5C and (j) 11.7 mg cm^−2^ at 0.2C. (k) Discharge/charge curves of the battery with a high S loading of 11.7 mg cm^−2^ at different cycles (E/S ratio = 8 µL mg^−1^).^97^ Copyright 2022 by the American Chemical Society.

Molecular interaction regulating polymer binder (PNAVS) was developed by Gong *et al.*^97^ A water-dispersible polymer binder was obtained by copolymerization of *N*-acryloyl glycinamide (NAGA) and 3-(1-vinyl-3-imidazolio)propanesulfonate (VIPS) to regulate molecular interactions in Li–S batteries. The tailored functional groups provided strong coordination with PS, increasing the binding energy to effectively suppress the shuttle effect and enhanced cycling stability. Simultaneously, the binder improved Li^+^ diffusion, accelerating redox kinetics. A schematic representation is shown in Fig. 14a–c. Self-discharge is a critical barrier to practical Li–S batteries. As shown in Fig. 4d, cells with the PNAVS binder exhibit an ultra-stable open-circuit voltage for over 3000 h, whereas PNAGA and PVDF show rapid initial decay, indicating weaker PS control. This stability surpasses most reported systems (Fig. 14e). In Li/Cu tests (Fig. 14f), PNAVS delivers a high and stable coulombic efficiency of 97.5% over 300 cycles at 1 mA cm^−2^, outperforming the other binders. Electrochemically, PNAVS enabled superior rate capability (Fig. 14g), delivering 1403, 1114, 986, 909, 823, 762 and 715 mAh g^−1^ at 0.1 to 4C, respectively. Long-term cycling at 1C (Fig. 14h) retains 647.8 mAh g^−1^ after 500 cycles, reflecting the strong PS adsorption and fast redox kinetics. Under practical conditions (E/S = 8 µL mg^−1^), PNAVS maintained stable cycling at high S loadings (Fig. 14i–k), 5.84 mg cm^−2^ sustains >190 cycles at 0.5C, 7.63 mg cm^−2^ delivered 5.91 mAh cm^−2^ over 80 cycles, 8.45 mg cm^−2^ achieved 9.53 mAh cm^−2^ at 0.1C. Even at 11.7 mg cm^−2^, it reached 12.21 mAh cm^−2^ at 0.1C and 10.64 mAh cm^−2^ at 0.2C. Overlapping charge–discharge plateaus (Fig. 14k) further confirmed the stable kinetics and cycling performance at ultrahigh S loading.

Some ion-conductive binders act like solid or gel-polymer electrolytes. They simultaneously provide mechanical binding and lithium-ion transport. These binders commonly incorporate plasticizers or ionic liquids to increase the ion mobility while preserving the structural integrity. Through intimate contact with S and carbon hosts, gel-type binders lower interfacial resistance and promote uniform lithium-ion distribution during electrochemical cycling. In addition to facilitating ion transport, many ion-conductive polymer binders contain polar functional groups that interact with lithium PS. These groups help to retain soluble intermediates within the cathode. This confinement suppresses the PS shuttle effect and enhances the coulombic efficiency of the battery. Collectively, improved ionic conduction and effective PS anchoring contribute to enhanced cycling stability and rate performance.^52,102,103^Table 5 summarizes the advantages, limitations and future directions of ion-conductive binders.

**Table 5:** Summary of the advantages, limitations and future research directions of ion-conductive polymer binders^93,104,105^

Advantages	Limitations	Future research directions
• Enhanced lithium-ion transport within the cathode	• Lower electronic conductivity	• Molecular design to enhance room-temperature ionic conductivity
• Improved active material utilization and redox kinetics	• Often requires additional conductive additives	• Development of structurally robust polymer frameworks
• Reduced ionic polarization, especially in thick electrodes	• Ionic conductivity sensitive to temperature, electrolyte composition and polymer crystallinity	• Optimization of ion transport pathways without compromising stability
• Improved interfacial contact and mechanical integrity	• Difficulty in balancing ionic transport, mechanical strength and chemical stability	• Integration with conductive networks or hybrid systems
• Enables high S loading cathodes		• Advancement towards high-energy-density and long-life Li–S batteries

### 4.6. Crosslinked and elastomeric binders

**Fig. 15:**
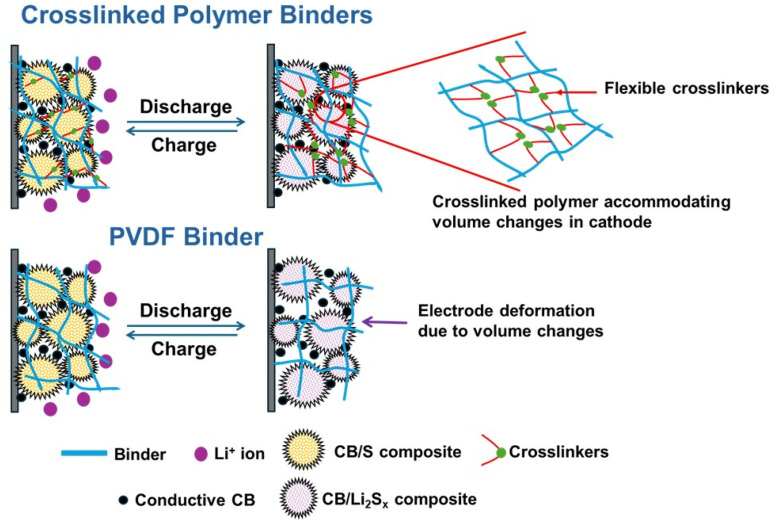
Schematic of the working mechanisms of a crosslinked polar polymer binder compared with that of a PVDF binder during battery cycling.

Crosslinked and elastomeric binders are studied extensively in Li-ion battery electrodes, specifically where volume expansion in electrodes is critical (*e.g.*, silicon, graphite, *etc.*). These binders have emerged as an important class of advanced binder materials for Li–S cathodes and Si anodes, specifically designed to address the severe mechanical instability caused by large volume changes during electrochemical cycling. Unlike linear polymer binders, crosslinked and elastomeric binders form three-dimensional networks that combine mechanical robustness with high elasticity. Crosslinked polymer binders are obtained by introducing covalent or dynamic crosslinking points into polymer matrices such as crosslinked chitosan sulfate, PAA, CMC, PEO or polyurethane.^80,106–111^ This unique architecture enables effective accommodation of the large and repeated mechanical stresses experienced by Li–S cathodes during cycling, Fig. 15. Through elastic stretching and reversible deformation, these binders absorb strain without fracturing. They thereby preserve electrode integrity and interparticle contact. The presence of crosslinking points serves as mechanical anchors that suppress irreversible polymer flow and prevent electrode disintegration, while flexible polymer segments allow reversible expansion and contraction. During de-lithiation, the elastic network contracts by restoring structural cohesion and maintaining intimate contact among S particles, conductive additives and the current collector. As a result, crosslinked and elastomeric binders significantly enhance the mechanical stability and long-term cycling performance.^80,106–112^

**Fig. 16:**
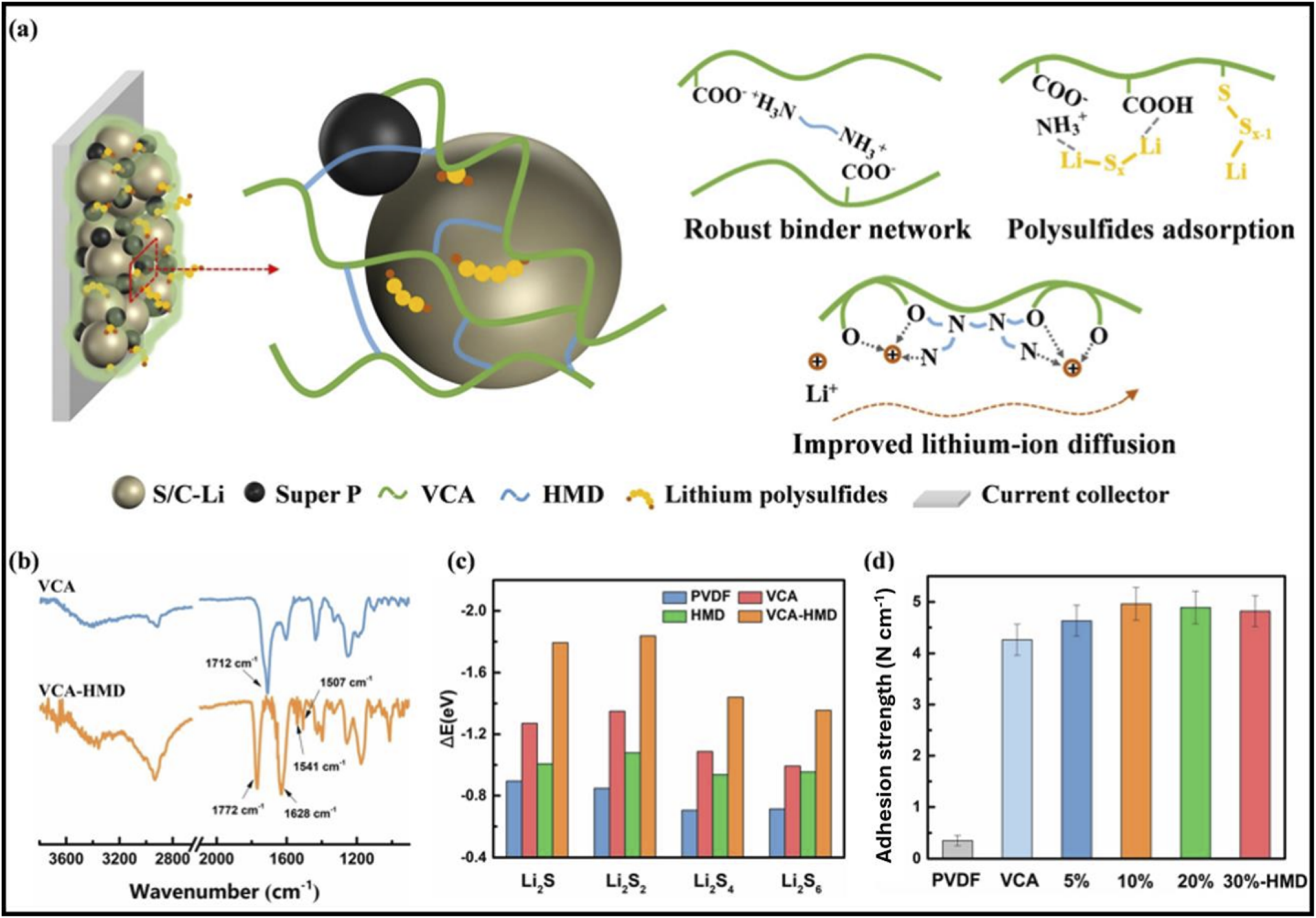
(a) Schematic of the design strategy for a crosslinked binder (VCA-HMD). (b) FTIR spectra of VCA and VCA-HMD. (c) Summary of the corresponding adsorption binding energies of various binders to LiPSs (selected species: Li_2_S, Li_2_S_2_, Li_2_S_4_ and Li_2_S_6_) calculated by DFT analysis. (d) Adhesion strength between two Al current collectors bonded with different binders measured by the peel test.^111^ Copyright 2021, Elsevier.

An *in situ* crosslinked binder (VCA-HMD) based on a chlorine-containing anionic polymer (VCA) and a diamine (HMD) has been developed for high-performance Li–S batteries.^111^ The optimized polymer network provides strong adhesion and controlled electrolyte swelling, while maintaining electrode integrity during prolonged cycling. The resulting network formed through –COOH/–NH_2_ interactions enhanced the mechanical integrity, while retaining unreacted –COOH groups for additional functionality. Polar O- and N-containing groups provided strong PS anchoring and improved Li^+^ transport, promoting faster electrochemical kinetics, Fig. 16a. The FTIR spectra confirmed successful crosslinking: the C=O peak at 1712 cm^−1^ split into two, and a new NH_3_^+^ peak appeared at 1507 cm^−1^, indicating the formation of carboxylate–ammonium interactions, as seen in Fig. 16b. Fig. 16c shows the DFT results, where VCA-HMD exhibited stronger binding energies toward Li_2_S_*x*_ (*x* = 1, 2, 4, and 6) than VCA, HMD and PVDF due to cooperative coordination with both O and N sites, effectively suppressing the shuttle effect. Optimized at a 1 : 10 ratio (VCA-10%HMD) the binder showed significantly higher adhesion strength than PVDF due to hydrogen bonding with the Al foil, ensuring robust electrode structure during cycling, Fig. 16d.

**Fig. 17:**
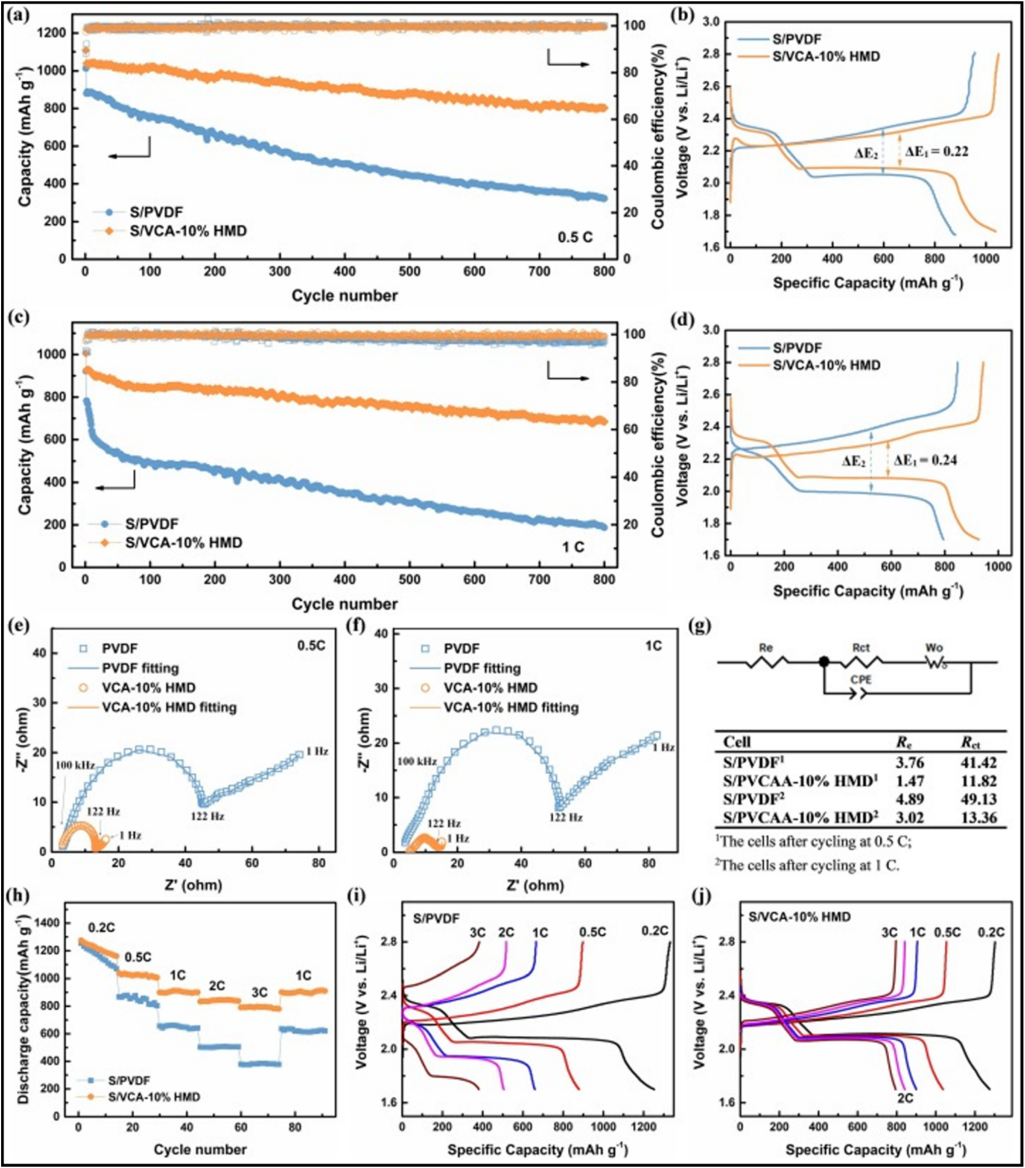
Electrochemical performance of S cathodes with different binders. Cycling stability and coulombic efficiency of S/PVDF and S/VCA-10% HMD cells at (a) 0.5C and (c) 1C. The initial discharge–charge profiles at (b) 0.5C and (d) 1C. Nyquist plot curves of the S/PVDF and S/VCA-10% HMD cells after 100 cycles at (e) 0.5C and (f) 1C. (g) Equivalent circuit models and fitting results for the S/PVDF and S/VCA-10% HMD cells after cycling. (h) Rate capacity of the S/PVDF and S/VCA-10% HMD cells. Discharge–charge profiles of the (i) S/PVDF and (j) S/VCA-10% HMD cells at different rates.^111^ Copyright 2021, Elsevier.

The electrochemical performance of the S/VCA-10%HMD cathode significantly surpasses that of S/PVDF at 0.5C, as seen in Fig. 17a. It delivered a higher initial capacity of 1038.1 mAh g^−1^*vs.* 880.4 mAh g^−1^ for PVDF, and retains 803.8 mAh g^−1^ after 800 cycles (77.4% retention, 0.028% decay per cycle) compared to only 323.1 mAh g^−1^ (36.7%, 0.079%) for PVDF. The voltage profiles in Fig. 17b show similar high-voltage plateaus (∼2.3 V). However, there is a prolonged low-voltage plateau (∼2.1 V) for VCA-10%HMD, indicating its enhanced PS anchoring and utilization. Fig. 17c and d shows battery performance at 1C, S/VCA-10%HMD maintained superior performance with an initial capacity of 926.9 mAh g^−1^ and 684.2 mAh g^−1^ after 800 cycles (73.8% retention, 0.033% decay) far exceeding PVDF (187.4 mAh g^−1^, 23.9%). It also showed higher coulombic efficiency and reduced polarization. Even at higher S loadings (3.0–4.0 mg cm^−1^), capacities of 722.4 and 582.5 mAh g^−1^ are achieved after 100 cycles at 1C. EIS analysis reveals much lower charge-transfer resistance for VCA-10%HMD (11.82 Ω at 0.5C, 13.36 Ω at 1C) when compared with PVDF (41.42 and 49.13 Ω), confirming improved charge transport, Fig. 17e–g. The rate capability further highlights its advantage with higher capacities at high rates, along with smaller voltage gaps for S/VCA-10%HMD compared to S/PVDF, as seen in Fig. 17h–j. These results demonstrate that the crosslinked binder enhances PS confinement, ion/electron transport and overall electrochemical stability.

Elastomeric binders, including SBR, nitrile rubber, silicone-based polymers and polyurethane elastomers, also offer high elasticity and excellent adhesion. Their rubber-like behavior allows continuous stretching and contraction during volume fluctuations without mechanical failure. Elastomeric binders also improve electrode adhesion to the current collector, reducing delamination during prolonged cycling. However, their relatively low polarity often needs chemical modification or blending with polar polymers to improve PS confinement. To enhance functionality, crosslinked and elastomeric binders are often functionalized with polar groups such as carboxyl, hydroxyl or sulfonate moieties.^74–77,110^ These groups provide chemical interactions with lithium PS, reducing dissolution and shuttle effects while retaining elastic properties. Such multifunctional designs allow binders to simultaneously stabilize the electrode structure and improve the electrochemical performance.^74–77,112^Table 6 summarizes the advantages, limitations and future directions of crosslinked polymer binders.

**Table 6:** Summary of the advantages, limitations and future research directions of crosslinked polymer binders^85,86,113,114^

Advantages	Limitations	Future research directions
• Accommodates large volume expansion during cycling	• Limited electronic and ionic conductivity	• Development of multifunctional binders with combined mechanical and electrochemical roles
• Enhanced mechanical durability and elasticity	• Requires integration with conductive additives or ion-conductive components	• Integration of PS confinement and enhanced charge transport
• Resists electrode cracking and pulverization	• Excessive crosslink density may reduce flexibility	• Advanced polymer design for optimized network structures
• Improves adhesion and structural integrity	• Can hinder electrode processing	• Scalable and practical processing strategies
• Suitable for high active material loading electrodes	• Challenge in balancing elasticity, chemical functionality and long-term stability	

### 4.7. Binder-free architecture

A key challenge in Li–S batteries comes from the physical and chemical instability of the cathode architectures that rely on polymer binders. While binders are required for maintaining electrode cohesion, conventional inert binders often induce electronic and ionic resistance, resulting in poor contact between the active materials and conductive networks. They are also mechanically unable to accommodate the large (≈80%) volume changes during cycling. These constraints are elevated in high S loading, thick electrodes aimed at practical energy densities. One of the strategies to overcome the limitations of binders in electrodes is using binder free three-dimensional architecture.^115^ This is one of the strategies that offers a powerful alternative to traditional polymer binders in Li–S cathodes by addressing intrinsic mechanical instability, electronic/ionic resistance and limited active material utilization. Self-standing films and ordered array films are some of the reported binder-free architectures for Li–S batteries. Such frameworks complement the advances in functional binder development and highlight the broader design space available for next-generation Li–S cathode engineering.^116–119^

**Fig. 18:**
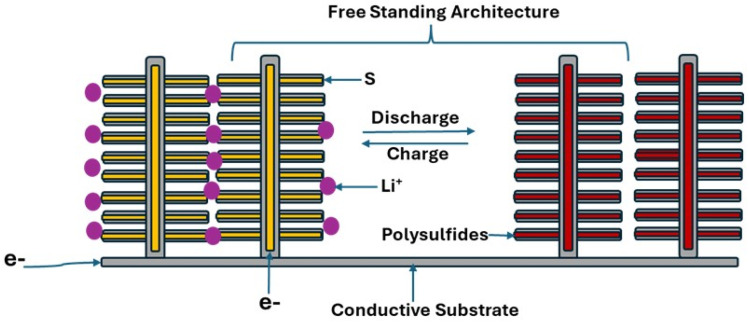
Schematic of the working mechanism of a binder-free Li–S cathode.

Self-standing binder-free cathode architectures are an effective way to overcome the key limitations of conventional Li–S electrodes. The binder-free architecture integrates mechanical support and charge-transport functionalities in a single continuous framework. Three-dimensional carbon network films offer interconnected electron pathways, large electrochemically active surface areas and sufficient void space to accommodate volume expansion during cycling. They also enable high S utilization even at high areal loadings.^120–122^ Incorporating polar inorganic components or conductive polymers into these networks further improves PS confinement and reaction kinetics through chemical adsorption and catalytic effects, while preserving electrical conductivity (Fig. 18). Overall, the rationally designed self-standing 3D carbon and hybrid architectures demonstrate that structural continuity, mechanical resilience and synergistic chemical functionality are critical design principles for achieving high-loading, high-energy-density and long-life Li–S cathodes.^123–125^

The ordered array binder-free cathode architecture demonstrates that structural alignment is as critical as material chemistry in Li–S batteries. By directly growing aligned carbon or polar inorganic nanostructures on current collectors, short and continuous electron pathways can be established. This architecture can enable uniform S distribution at high loadings, and create nanoscale reservoirs that physically and chemically confine PS. The resulting constructive collaboration between the rapid charge transport, effective shuttle suppression and mechanical robustness can deliver better rate capability and long-term cycling stability even with high S loading. These findings highlight the ordered array design as a powerful strategy for simultaneously achieving high energy density and high-power performance.^126–131^

**Fig. 19:**
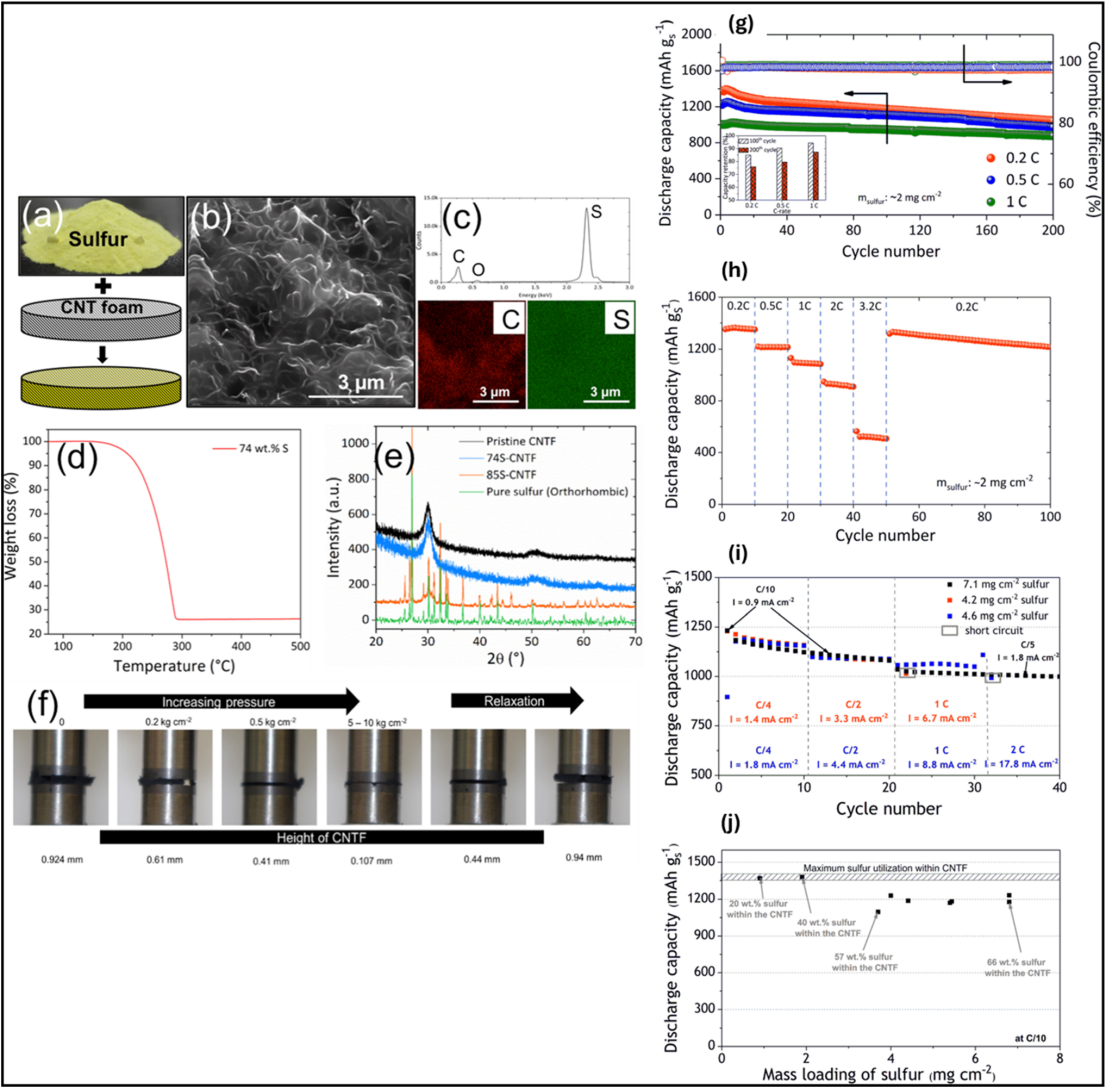
(a) Schematic of S impregnation onto CNTF. (b) SEM image of the S-impregnated CNTF. (c) EDX spectrum and mapping of S and carbon species in the S-impregnated CNTF. (d) TG profile of the 74 wt% S-containing CNTF (74S-CNTF electrode). (e) XRD patterns of pristine and S-impregnated CNTFs (intensities of the spectra from 85S-CNTF and pure S were reduced by one-third and increased by five times, respectively, for the sake of comparison). (f) Variation in the thickness of CNTF at different pressures reasonably present in coin cells. (g) Cycling performances of the 33S-CNTF electrode at 0.2C, 0.5C and 1C. (h) Rate performance of the Li–S cell with 33S-CNTF electrode. (i) Rate tests of the S-CNTF at different S loadings. At high current rates of 7–18 mA cm^−2^, lithium dendrites grow extremely fast and cause short circuit of the cell. (j) Effect of S mass loading on the initial discharge capacity at a current rate of C/10.^116^ Copyright 2018, Elsevier.

Ummethala *et al.*^116^ developed a 3D carbon nanotube foam (CNTF) that is free-standing, flexible and binder-free by fabricating a simple single-step process. This process avoided conventional solvent-based assembly routes such as vacuum infiltration and post-treatment. CNTF electrodes were prepared by just punching out disks from the as-synthesized foam material, followed by solvent-free S impregnation *via* uniform S coating and heating at 155 °C for 1 h under Ar, Fig. 19a. This simple, scalable approach avoids complex vacuum infiltration and enables high-throughput production with minimal impurities. Fig. 19b and c shows the SEM and EDXS mapping, respectively. These results confirm the uniform S distribution within the 3D CNT network without agglomeration, preserving the porosity for electrolyte penetration and accommodating volume changes. TGA (Fig. 19d) shows the presence of ≤74 wt% S loading, while XRD (Fig. 19e) indicates that there are no crystalline S peaks, confirming the presence of the highly dispersed amorphous S. Despite the initial high porosity (∼96% void volume at 1000 µm thickness), mild compression (<1 bar) reduces the thickness to ∼150 µm, decreasing the void space to <50% while maintaining structural integrity. The foam can shrink to ∼11.6% of its original volume under pressure, enabling tunable porosity and significantly improved volumetric energy density, Fig. 19f. The 33S-CNTF electrode (∼2 mg cm^−2^) demonstrates excellent cycling stability across different rates (Fig. 19g). Initial capacities at 0.2, 0.5 and 1C are 1379, 1225 and 1004 mAh g^−1^, respectively, and remained high after 200 cycles at 1046, 975 and 877 mAh g^−1^. This corresponded to capacity retentions of 75.8%, 79.6% and 87.3%. Capacity fading rates were low at 0.120%, 0.102% and 0.063% per cycle, while coulombic efficiency improved from 97.8% to 99% with increasing rate, reflecting reduced PS shuttling. Rate capability tests (Fig. 19h) showed stable capacities of 1350, 1215 and 1084 mAh g^−1^ at 0.2, 0.5 and 1C and still 909 and 507 mAh g^−1^ at 2 and 3.2C. When the rate returned to 0.2C, a high reversible capacity of 1318 mAh g^−1^ is recovered, confirming the strong reversibility. Even at high current densities of up to 17 mA cm^−2^ and S loading of 4.6 mg cm^−2^, capacities near 1000 mAh g^−1^ are achieved (Fig. 19i). However, cell failure at extreme rates is attributed to lithium dendrite growth, rather than cathode limitations. Capacity dependence on S loading (Fig. 19j) was evaluated, which showed ∼1400 mAh g^−1^ at <2 mg cm^−2^ and decreased to ∼1200 mAh g^−1^ at ∼4 mg cm^−2^ (57 wt%) due to increased insulation, but this trend remained stable up to loadings of 7 mg cm^−2^, highlighting the excellent conductivity and PS confinement of the CNTF structure. Table 7 summarizes the advantages, limitations and future directions of binder-free architecture concept.

**Table 7:** Summary of the advantages, limitations and future research directions of binder-free architectures^115,120,124,130^

Advantages	Limitations	Future research directions
• Provides continuous electronic and ionic transport pathways	• Difficult and costly to scale up for industrial production	• Development of scalable and cost-effective fabrication methods
• Reduces polarization and enhances redox kinetics	• Complex fabrication processes	• Incorporation of polar or catalytic components for stronger PS confinement
• Enables uniform S distribution and utilization	• Porous carbon systems rely mainly on weak physical PS confinement	• Hybrid architecture combining physical and chemical trapping mechanisms are required
• Offers inherent mechanical robustness without polymer binders	• Insufficient suppression of PS dissolution under high S loading or long-term cycling	• Optimization for practical conditions (high loading, lean electrolyte)
• Better accommodates volume changes during cycling	• Gradual capacity decay over extended operation	• Translation into commercially viable Li–S battery systems

Table 8 summarizes the electrochemical performance of Li–S batteries employing the various polymer binder systems discussed in this review. Only studies that demonstrated longer cycle life and improved performance (capacity and cycle life) at moderate to high C-rates under practically relevant active material loadings are included in Table 8 to ensure meaningful and comparative evaluation. Although all advanced binder systems discussed in this review aim to improve Li–S battery performance, they achieve this through different mechanisms and involve distinct trade-offs. For example, functional polar binders primarily suppress the PS shuttle through strong chemical interactions due to the presence of active functional groups. In contrast, conductive and ion-conductive binders enhance electron and Li^+^ transport, thereby improving the S utilization and rate capability. Meanwhile, supramolecular, dynamic, crosslinked, and elastomeric binders focus on maintaining electrode integrity by accommodating large-volume changes and preserving interparticle contact during cycling. Additionally, bio-based binders offer further advantages in sustainability and scalable processing. Nevertheless, for practical Li–S batteries, no single functionality is sufficient. Thus, an effective binder design requires balancing the PS regulation, charge transport, mechanical robustness, and compatibility with high S loading and lean electrolyte under practical operating conditions. Consequently, recent research has increasingly shifted toward multifunctional binder systems that integrate several of these properties within a single material platform.

**Table 8:** Summary of the battery performance obtained using different types of polymer binders discussed in this review^a^

Binder type	Total binder %	S loading mg cm^−2^	Measured C-rate	Electrolyte volume per mg of S	1st/2nd Cycle capacity in mAh g^−1^	% Capacity retention	Obtained cycles	Reference number
PVDF	10	7.86	2	9 µL	900	44	600	61
	10	7.42	1	8.5 µL	700	50	500	64
Conductive binders	10	—	0.5	—	1097	73	250	47
	10	4.7	0.5	20	950	80	500	42
Supramolecular and dynamic binders	10	7.86	2	9.0 µL	960	59	600	61
	15	5.6	1	5.8 µL	900	78	800	62
	10	7.42	1	8.5 µL	933	80	500	64
Functional polar polymer binders	10	3.5	2	—	1089	62	300	79
	10	11.0	0.1	6.4 µL	978	68	100	83
Bio-based and sustainable binders	10	4.2	2	8.0 µL	628	93	400	88
	10	3.0	0.2	21.5 µL	1629	42	1000	89
Ion-conductive polymer binders	10	11.7	1	8.0 µL	900	72	500	97
	10	3.7	1	7.0 µL	1300	71	100	103
Crosslinked and elastomeric binders	10	7.0	1	—	750	85	1000	110
	10	3.0	1	—	927	74	800	111
	10	8.7	1	8.2 µL	907	76	600	113
Binder-free architecture	NA	2.0	1	—	1004	87	200	116
	NA	4.1	2	5.0 µL	621	77	500	130

a Direct comparisons of binder performance across reported studies should be made with caution due to variations in S loading, S/electrolyte ratio, current density, cycling protocols, and cell configurations. Accordingly, Table 8 is intended to highlight general performance trends rather than provide a rigorous quantitative ranking.

Despite significant progress in binder development, assessing their practical applicability remains challenging because several key cell-level parameters are not consistently reported in the literature. Metrics such as the negative-to-positive capacity ratio, electrode density, binder content, and validation in pouch-cell configurations are frequently omitted, limiting direct evaluation of commercial relevance. Consequently, many binder systems that demonstrate excellent performance in laboratory-scale coin cells have yet to be validated under realistic operating conditions. Future studies should adopt standardized reporting practices and include these practical metrics to enable more meaningful comparisons and accelerate the translation of advanced binder technologies toward commercially viable Li–S batteries.

## 5. Conclusion

In this review, we discussed multiple approaches that were explored for the development of multifunctional binder systems. The discussed functional polar polymer binders effectively suppress the PS shuttling through strong chemical interactions. Crosslinked and elastomeric binder networks provide essential mechanical resilience against the repeated volume changes of the Li–S cathodes. Electron conductive and ion-conductive binders further enhance active material utilization by establishing continuous electrons and lithium-ions transport pathways. These pathways provided by the binder results in reduced polarization and improved rate capability. As seen from studies, supramolecular and dynamic covalent binders bring in self-healing and stress-relaxation mechanisms that help maintain electrode integrity during prolonged cycling. In parallel, bio-based and water-processable binders address increasing demands for sustainability, thermal stability, safety and scalable manufacturing. The binder-free cathode architecture has shown promising results by offering mechanical reinforcement, effective PS confinement and catalytic functionality. Similar advancements have also been observed in the Li-ion battery technology, where binder systems have evolved from inert mechanical components to multifunctional materials capable of improving the electrode integrity, ion transport and interfacial stability. The successful development of advanced binders in Li-ion batteries demonstrates the importance of polymer engineering in achieving high-performance and durable energy storage systems.^132–135^ These insights can further guide the rational design of next-generation multifunctional binders for practical Li–S battery applications.

Achieving an optimal balance between mechanical strength, chemical functionality, ionic/electronic conductivity and long-term chemical stability remains a challenge, particularly under high active material loading and lean electrolyte conditions. In addition, issues related to the cost, processability and compatibility with industrial electrode fabrication must be carefully considered. However, the future is expected to move towards designing multifunctional binders. Binders are expected to have elasticity, chemical anchoring, self-healing capability properties and transport enhancement within a single material platform.

## 6. Future research for Li–S cathode binder development

Although each binder type discussed in this review has its own advantages, they have their limitations that stop them from being used in Li–S batteries on a commercial scale. Future binder development for Li–S batteries should focus on multifunctional systems that simultaneously address electrochemical, mechanical and manufacturing challenges. Molecular-level design strategies linking polymer structure, functional groups, crosslinking density and interfacial interactions with battery performance will be essential for rational binder engineering. Next-generation binders are expected to integrate PS anchoring, mechanical elasticity, self-healing behavior and ionic/electronic conductivity within a single platform to simplify the cathode architecture and improve stability.

Greater emphasis should also be placed on practical operating conditions, including high S loading, lean electrolyte ratios, high current densities and extended cycling stability. In parallel, sustainable and scalable binder systems based on bio-derived materials, water-processable chemistries and industry-compatible fabrication methods will become increasingly important. Future progress will further benefit from co-engineering binder–electrode–electrolyte interfaces and integrating computational modelling, high-throughput experimentation and data-driven approaches to accelerate binder discovery and optimization.

## Author contributions

Pranjalee Ghosh conducted the literature survey, prepared the figures and tables, and contributed to the writing and revision of the manuscript. Manu U. M. Patel conceived and supervised the review, developed the overall structure and direction of the manuscript, wrote the initial draft, coordinated the writing process, and finalized the manuscript.

## Conflicts of interest

The authors declare that they have no known competing financial interests or personal relationships that could have appeared to influence the work reported in this paper.

## Data Availability

This is a review article and does not report any new experimental or computational data. All data supporting the findings of this study were derived from previously published articles, which have been appropriately cited in the reference list. Any additional information related to this review can be obtained from the corresponding author upon reasonable requests.
